# Cell Uptake of Steroid-BODIPY Conjugates and Their Internalization Mechanisms: Cancer Theranostic Dyes

**DOI:** 10.3390/ijms24043600

**Published:** 2023-02-10

**Authors:** Ana F. Amendoeira, André Luz, Ruben Valente, Catarina Roma-Rodrigues, Hasrat Ali, Johan E. van Lier, Fernanda Marques, Pedro V. Baptista, Alexandra R. Fernandes

**Affiliations:** 1 Associate Laboratory i4HB, Institute for Health and Bioeconomy, NOVA School of Science and Technology, 2819-516 Caparica, Portugal; 2 UCIBIO—Applied Molecular Biosciences Unit, Department of Life Sciences, NOVA School of Science and Technology, 2819-516 Caparica, Portugal; 3 Department of Nuclear Medicine and Radiobiology, Faculty of Medicine and Health Sciences, University of Sherbrooke, Sherbrooke, QC J1H5N4, Canada; 4 Centro de Ciências e Tecnologias Nucleares, Departamento de Engenharia e Ciências Nucleares, Instituto Superior Técnico, Universidade de Lisboa, Estrada Nacional 10, km 139.7, 2695-066 Bobadela, Portugal

**Keywords:** estradiol-BODIPY conjugates, androgen-BODIPY conjugates, fluorescence imaging, photosensitizers, receptor-mediated cell uptake, photodynamic therapy

## Abstract

Estradiol-BODIPY linked via an 8-carbon spacer chain and 19-nortestosterone- and testosterone-BODIPY linked via an ethynyl spacer group were evaluated for cell uptake in the breast cancer cell lines MCF-7 and MDA-MB-231 and prostate cancer cell lines PC-3 and LNCaP, as well as in normal dermal fibroblasts, using fluorescence microscopy. The highest level of internalization was observed with 11β-OMe-estradiol-BODIPY **2** and 7α-Me-19-nortestosterone-BODIPY **4** towards cells expressing their specific receptors. Blocking experiments showed changes in non-specific cell uptake in the cancer and normal cells, which likely reflect differences in the lipophilicity of the conjugates. The internalization of the conjugates was shown to be an energy-dependent process that is likely mediated by clathrin- and caveolae-endocytosis. Studies using 2D co-cultures of cancer cells and normal fibroblasts showed that the conjugates are more selective towards cancer cells. Cell viability assays showed that the conjugates are non-toxic for cancer and/or normal cells. Visible light irradiation of cells incubated with estradiol-BODIPYs **1** and **2** and 7α-Me-19-nortestosterone-BODIPY **4** induced cell death, suggesting their potential for use as PDT agents.

## 1. Introduction

Theranostics refers to the pairing of diagnostic biomarkers with therapeutic agents that share a specific target in diseased cells or tissues. In the case of cancer, it is desirable to develop agents that can diagnose the disease and can also be used for treatment. Recently, it has been reported that combining a steroid hormone with a BODIPY dye shows binding affinity for the estrogen receptor. The characteristic spectroscopic properties, narrow absorption and emission bands, strong absorption coefficients in the visible range (470–530 nm), high fluorescence quantum yields, excellent photostability, moderate redox potentials, low triplet-state formation, and singlet oxygen yield of BODIPY dyes (4,4,-difluoro-4-bora-3a,4a-diaza-s-indacene) suggest that they should be studied as theranostic agents [1,2,3,4]. These properties can be modulated by appropriate chemical tuning of the chromophore [1,5]. BODIPY derivatives have found a broad range of applications as chemo-sensors [6,7,8,9], logic gates [10,11,12], energy transfer cassettes [13,14,15], and sensitized solar cells [16,17,18,19]. They have also been used as fluorescent agents to label a variety of different ligands, including proteins [20,21], DNA [22,23], carbohydrates and lipids [24,25], peptides [26,27], and steroid hormones [28,29,30]. These properties have led researchers to explore their biomedical applications as photosensitizers in photodynamic therapy (PDT) [31,32,33], positron emission tomography (PET) [34,35,36], and fluorescence imaging [37,38].

Steroid hormone receptors have emerged as particularly attractive targets for molecular imaging due to their role in promoting the growth of hormone-dependent breast, ovarian, uterine, and prostate cancers [39]. Ligands for the imaging of breast and prostate cancer have been advanced either as fluorescent dyes for optical imaging or positron-emitting radiopharmaceuticals for PET imaging [40,41]. Steroid hormones bind to their cognate nuclear receptors, which are involved in the regulation of complex gene networks, with high affinity [42]. 17β-Estradiol (E2) is a key regulator of growth, differentiation, and function in a wide array of target tissues, including the male and female reproductive tracts, mammary glands, and skeletal and cardiovascular systems [43,44]. The effects of E2 are mediated through intracellular estrogen receptors (ERα and ERβ) [44,45]. Many cells throughout the human body contain ER, but its levels are higher in premalignant and malignant breast lesions as opposed to normal tissue [46]. “ER-positive” (ER+) breast cancer is the most common cancer subtype, and hormone therapy and drugs that target and block these receptors are extensively used for systemic treatment in adjuvant and metastatic therapeutic settings [47,48]. Most types of breast cancer overexpress hormone receptors, making these receptors interesting targets for the in vivo imaging of ER densities in human breast cancer [47,48]. Thus ER imaging can potentially be used for breast cancer screening, staging, and response evaluation and for guiding therapies [49,50]. Many techniques have been developed to detect ligand–ER interactions, including PET [51,52] and fluorescence imaging [53,54].

The progesterone receptor (PgR) is strongly associated with disease prognosis and therapeutic efficacy in hormone-related diseases such as endometriosis, breast, ovarian, and uterine cancers [55], while androgen receptors (AR) play a fundamental role in the development and survival of male reproductive tissues, such as the prostate [56]. Testosterone is the most abundant androgen in men [57]. Low testosterone levels lead to fatigue and erectile dysfunction, and high levels have been linked to a variety of diseases, particularly prostate cancer [57], which is the most common cancer among men [58]. PgR levels in breast tumors and AR in prostate tumors also provide important prognostic information for the detection and treatment of hormone-responsive neoplasms. In the case of breast cancer, positive PgR levels have shown to be more predictive of tumor response to hormonal therapy than ER levels [49,59]. This suggests that a progestin-based agent for breast tumor imaging might be preferable for patients on hormonal therapy. Several radiolabeled progestins have been reported [60,61]; however, they have a low specific activity and low affinity for the PgR, preventing selective uptake by PgR-rich target tissues in vivo.

**Scheme 1:**
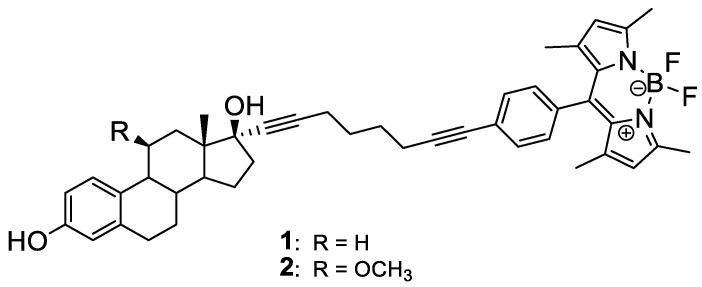
Structures of estradiol-BODIPY conjugates.

**Scheme 2:**
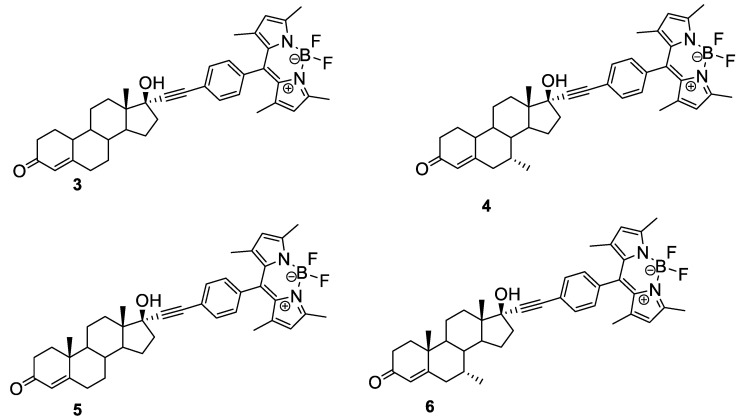
Structures of 19-nortestosterone- and testosterone-BODIPY conjugates.

Recently, it has been shown that among a series of E2-BODIPY conjugates, the highest receptor binding affinity (RBA) for ERα was observed in conjugate **1**, featuring a linear eight-carbon spacer chain (Scheme 1), whereas the parent conjugate lacking the spacer chain showed little affinity for the ER [62]. A series of estrogen-, 19-nortestosterone-, and testosterone-BODIPY conjugates were subsequently prepared, providing a platform of potential receptor-based fluorescence ligands for imaging breast and prostate cancer [63]. The 11β-OMeE2 conjugate **2** is of particular interest, since it has been reported that the 11β-OMe substitution of estradiol facilitates in vivo localization in receptor-rich tissues [51,61]. In view of the significant binding affinities (AR and PgR) reported for radio-iodinated 17α-ethynyl-19-nortestosterone and 17α-ethynyl-testosterone [64], new 19-nortestosterone-(**3**, **4**) and testosterone-(**5**, **6**)-BODIPY conjugates (Scheme 2), which were previously characterized, were included in this study (see the Materials and Methods section).

In this study, the internalization of BODIPY conjugates **1**–**6** in breast and prostate cancer cells was evaluated by fluorescence microscopy. Their mechanisms of cellular uptake were studied by blocking common cell internalization pathways (active transport vs. passive transport and endocytosis), and intracellular tracking of the conjugates was performed. Finally, a combined therapy was studied via the incubation of cancer cells with the BODIPY conjugates followed by visible light irradiation to assess the conjugates’ potential for PDT.

## 2. Results

A diverse group of tumors are acknowledged as hormone-dependent and, in such cases, an overexpression of hormone receptors is generally observed [65]. The classification and therapeutics of breast cancer are mainly centered on the presence of three receptors—ER, PR, or human epithelial receptor 2 (HER2) [66]. The presence of AR is also critical for prostate carcinogenesis and therapeutics [67]. In this study, we evaluated the steroid-BODIPY conjugates **1**–**6** as theranostics (optical tumor imaging and PDT). Two breast cancer cell lines, MCF-7 (ER- and AR-responsive) [66,68] and MDA-MB-231 (mostly reported as ER- and AR-non-responsive) [66,69]; two prostate cancer cell lines, PC-3 (AR-non-responsive and ER-responsive) [70,71] and LNCaP (AR-responsive and ER-non-responsive) [72]; and human primary dermal fibroblasts were selected, the latter serving as a hormone-independent cell line for control purposes (Figure S1).

### 2.1. Cell Uptake and Trafficking of Steroid-BODIPY Conjugates

Taking advantage of the fluorescence properties of the BODIPY probes, with conjugates presenting an UV/Vis absorption spectrum that ranges from 500 to 710 nm and fluorescence emission ranging from 520 to 700 nm [63], the internalization and intracellular tracking of conjugates **1**–**6** were assessed by fluorescence microscopy. The cells were incubated with 50 µM of BODIPY conjugates **1**–**6** for 45 min, 2 h, and 6 h, and the cells were imaged by fluorescence microscopy to quantify their intracellular fluorescence.

#### 2.1.1. Steroid-BODIPY Conjugates in Breast Cancer Cells

**Figure 1:**
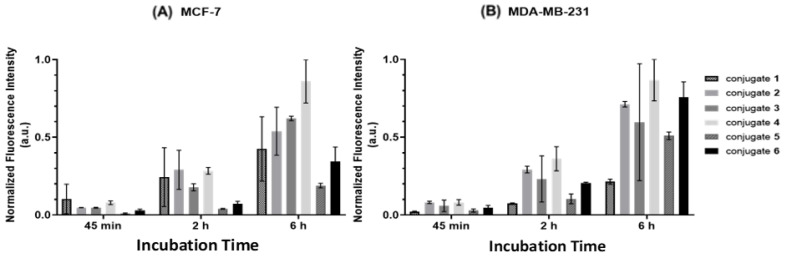
Normalized fluorescence intensities of MCF-7 (**A**) and MDA-MB-231 cells (**B**) upon incubation with steroid-BODIPY conjugates **1**–**6**.

The existence of ER-responsive breast cancers [73] opens up the field to the use of hormone derivatives and conjugates for cancer treatment. MCF-7, an ERα-responsive breast cancer cell line [66], is a useful in vitro model for the assessment of cancer responses to hormone therapy using estradiol derivatives. The quantification of cell fluorescence after incubation with estradiol-derived conjugates **1** and **2** and the 19-nortestosterone- or testosterone-derived conjugates **3**–**6** revealed that the fluorescence intensity increased over the course of the experiment (Figure 1A), as previously observed with other BODIPY conjugates [74,75]. Despite not presenting the highest fluorescence intensity at 6 h, the fluorescence levels of the estrogen-derived conjugates **1** and **2** are equivalent to those of the androgen conjugates **3**–**6** at 2 h (Figure 1A and Figure S2A). This might be due to increased cellular permeability or an affinity for the dyes in the first hours of incubation [74,76]. BODIPY conjugate **2** shows a higher fluorescence than conjugate **1**. In addition to the long spacer chain that improves ER binding, conjugate **2** features a polar methoxy group at C-11 (Scheme 1) that may facilitate in vitro localization in the target cells and increase the target/non-target ratios, as previously observed in related estrogens in vivo [77].

After 6 h of incubation, conjugate **4** presented the highest fluorescence intensity (Figure 1A and Figure S2B), which might be explained by the presence of AR in the MCF-7 cells [68]. The higher cell uptake of **4** as compared to **3**, lacking the 7αMe-substituent, may also reflect differences in lipophilicity between the two analogs. Conversely, the testosterone-BODIPY conjugate **5**’s associated fluorescence levels are the lowest, which can be explained by the low affinity for AR of testosterone derivatives as compared to their 19-nortestosterone analogs [64,78,79]. Indeed, the levels of fluorescence for testosterone conjugates **5** and **6** were found to be the lowest over time (Figure 1A and Figure S2B).

A specific subtype of breast cancer lacking nuclear ER, PR, and HER2 expression has been defined as triple-negative breast cancer (TNBC) and accounts for 10–20% of all breast cancer cases [80]. MDA-MB-231 is a TNBC cell line that has low expressions of both ERα and AR [66,69,81]. The fluorescence levels of MDA-MB-231 upon 6 h of incubation show that conjugates **1**–**6** were all internalized (Figure 1B). The lower fluorescence intensities were observed after 2 h and 6 h for the estradiol-based conjugate **1** and for the testosterone-based conjugate **5** (Figure 1B and Figure S3). The 11β-OMe-E2 conjugate **2** and 7α-Me-testosterone conjugate **6** showed a strong increase in cell uptake, likely reflecting changes in the overall lipophilicity and cell-membrane-penetrating properties of the conjugates. Despite the BODIPY conjugates’ internalization in MDA-MB-231 cells, the intracellular fluorescence images (Figure S3) suggest a different type of accumulation of the conjugates, namely, in intracellular vesicles.

#### 2.1.2. Steroid-BODIPY Conjugates in Prostate Cancer Cells

**Figure 2:**
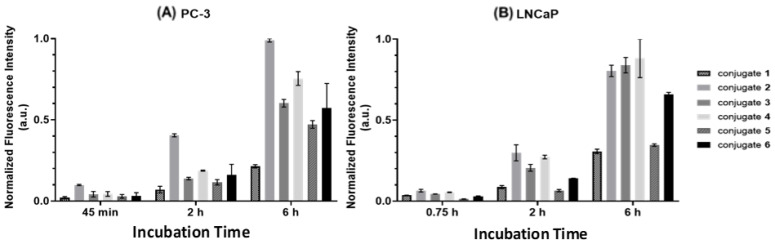
Normalized fluorescence intensity of PC-3 (**A**) and LNCaP (**B**) cells upon incubation with steroid-BODIPY conjugates **1**–**6**.

The PC-3 cell line is one of the most frequently used models for androgen-independent prostate cancers due to its very low or lack of AR expression [71]. This prostate cancer cell line is an ER-positive model [70], whereas the expression of the classical ER (ERα and ERβ) is still controversial [82]. The fluorescence analysis of PC-3 cells after exposure to conjugates **1**–**6** revealed that both the androgen and estradiol conjugates were internalized, with increasing fluorescence levels observed for all the conjugates during prolonged incubation times (Figure 2A and Figure S4). Conjugate **2** showed much higher cellular fluorescence as compared to conjugate **1** after 6 h (Figure 2A and Figure S4A). Indeed, when compared to all the BODIPY conjugates, conjugate **2** showed the highest level of intracellular fluorescence (Figure 2A), which is interesting, as this is an ER+ model. However, the cellular uptake of the androgen-BODIPY analogs, particularly the 19-norestosterone conjugates **3** and **4**, was higher than that observed for the estradiol-BODIPY conjugate **1** (Figure 2A and Figure S4), despite the AR-negative status of PC-3 cells. Among the androgen conjugates **3**–**6**, conjugate **4** has higher cellular fluorescence levels, reflecting structural alterations in the steroid skeleton, promoting increased uptake, which is in line with the results obtained for breast cancer cells (Figure 1). As previously stated for MDA-MB-231 cells (Figure S3), the fluorescence images of PC-3 cells (Figure S4) suggest an accumulation of the conjugates in the intracellular vesicles.

The LNCaP prostate cancer cell line is the most widely used AR-positive cell line due to its significant expression levels of AR [72] and the fact that it is an ER-negative cell line. As observed in the other cell lines, the intensity of green fluorescence increased with the exposure time for all the steroid-BODIPY conjugates studied (Figure 2B and Figure S5). 19-Nortestosterone-BODIPY conjugates **3** and **4**, as well as 11β-OMeE2 conjugate **2**, showed higher cellular fluorescence levels as compared to conjugate **1** or testosterone derivatives **5** and **6** (Scheme 2). Once again, as observed in the other cell lines, 19-nortestosterone conjugates **3** and **4** demonstrated a fast uptake pattern. Since this cell line is ER-non-responsive, as in the case of MDA-MB-231, the high cellular uptake observed for conjugate **2** appears to involve a mechanism of internalization independent of the ER-responsive status (Figure 1B and Figure 2B).

### 2.2. Internalization of Steroid-BODIPY Conjugates in 2D Co-Culture

Co-culture systems are often applied to elicit some in vivo conditions, allowing a diverse range of cell types to be cultured together so as to investigate the influence of one type of culture system on the other [83,84]. Co-cultures of cancer cells (MDA-MB-231 or PC-3) and fibroblasts (normal cells) were performed in a cell proportion of 1:1 to correlate the levels of BODIPY conjugates in cancer and normal cells and to elucidate the conjugate specificity for cells expressing the respective steroid receptors. As our previous results show, internalization, per se, appears to be independent of the receptor status (Figure 1, Figure 2 and Figure S7).

**Figure 3:**
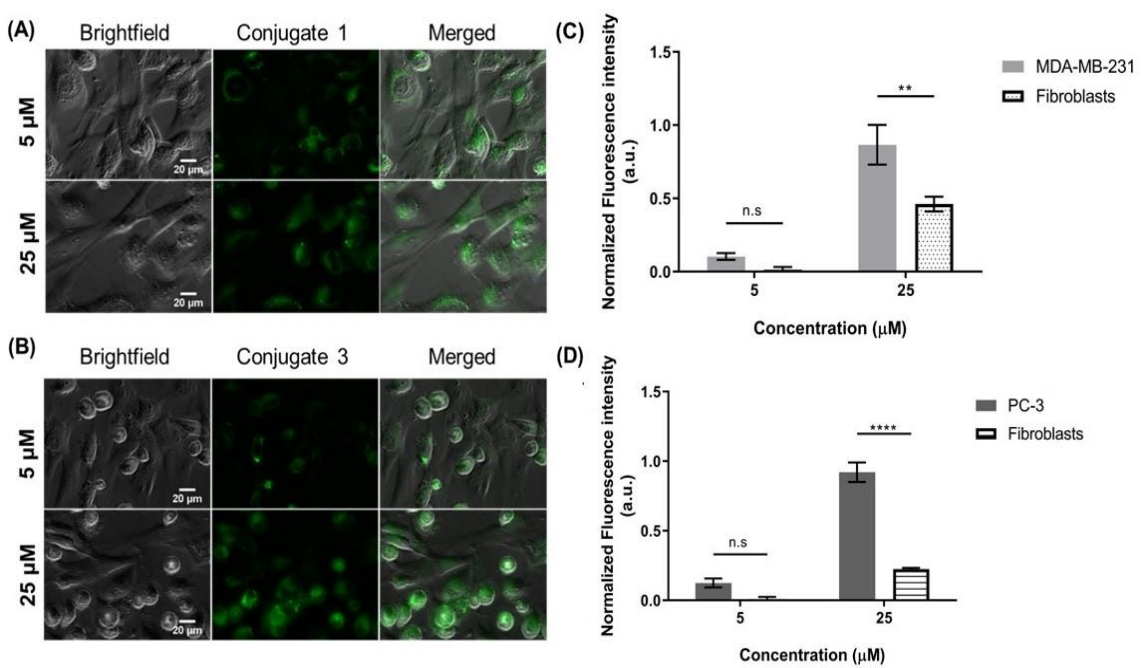
Internalization of E2-BODIPY conjugate **1** and 19-nortestosterone-BODIPY conjugate **3** in MDA-MB-231/fibroblast or PC-3/fibroblast co-cultures. Co-cultures were incubated for 6 h with 0.25% (*v*/*v*) DMSO (control) or 5 μM and 25 μM of steroid-BODIPY conjugate (conjugate 1 for MDA-MB-231: fibroblast co-culture and conjugate **3** for PC-3: fibroblast co-culture). (**A**) MDA-MB-231: fibroblasts and (**C**) PC-3. The fibroblast cells were visualized by an inverted microscope, and the fluorescence intensity was quantified for each co-culture, (**B**,**D**), respectively. The fluorescence intensity of the cells was corrected for background fluorescence and normalized with vehicle (DMSO). The statistical significance was evaluated by the two-way ANOVA method (** *p* ≤ 0.01, **** *p* ≤ 0.0001, n.s—not significant).

The internalization of E2 BODIPY conjugate **1** in the co-culture of MDA-MB-231 cells and fibroblasts (Figure 3A,C) revealed increasing internalization levels with the increased conjugate concentration. The normalized fluorescence levels were considerably higher in the MDA-MB-231 cells than in the fibroblasts for both of the concentrations tested, 5 µM and 25 µM (Figure 3C), which suggests a higher specificity of conjugate **1** to the breast cancer cells than to the fibroblasts, which is important in the context of cancer therapy. Additionally, MDA-MB-231 cells are ER non-responsive [66] and, therefore, the internalization of E2 conjugate **1** is likely independent of the presence of ER.

The internalization of 19-nortestosterone-BODIPY conjugate **3** in the PC-3 cells and the fibroblast co-culture was similar to that observed for the co-culture of MDA-MB-231 cells and fibroblasts with E2 BODIPY conjugate **1** (Figure 3). Increasing the concentration of conjugate **3** yielded an increase in the internalization levels (Figure 3B,D). With lower concentrations, the internalization of conjugate **3** in the fibroblasts was almost absent, indicating that it is more specific for cancer cells than for normal cells (Figure 3D), which, again, is a positive issue in efforts to improve cancer cell targeting. It is interesting to note that BODIPY conjugates can internalize fibroblasts in the absence of cancer cells (Figure S7). However, the fibroblasts in co-culture with cancer cells exhibited a significant decrease in, or absence of, fluorescence intensity (Figure 3C,D). These results allow us to conclude that steroid-BODIPY conjugates are more specific for cancer cells than for healthy cells when both types of cells co-exist (as in the tumor microenvironment). It is interesting to note that the internalization of E2-BODIPY conjugate **1** in the fibroblasts was higher when they were co-cultured with MDA-MB-231 cells, an ER-non-responsive cell line, as compared to the internalization of 19-nortestosterone-BODIPY conjugate **3** in PC-3 cells, an AR-non-responsive cell line (Figure 3A,B). Indeed, conjugate **1** showed the lowest internalization in the MDA-MB-231 cells among all the conjugates tested (Scheme 1 and Scheme 2), enabling its uptake by the fibroblasts (Figure 3C), while in the case of 19-nortestosterone conjugate **3**, its high uptake by the PC-3 cells coincided with a lower uptake by the normal cells (Figure 3D).

### 2.3. Internalization of Steroid-BODIPY Conjugates in the Presence of Endocrine Disruptors

**Figure 4:**
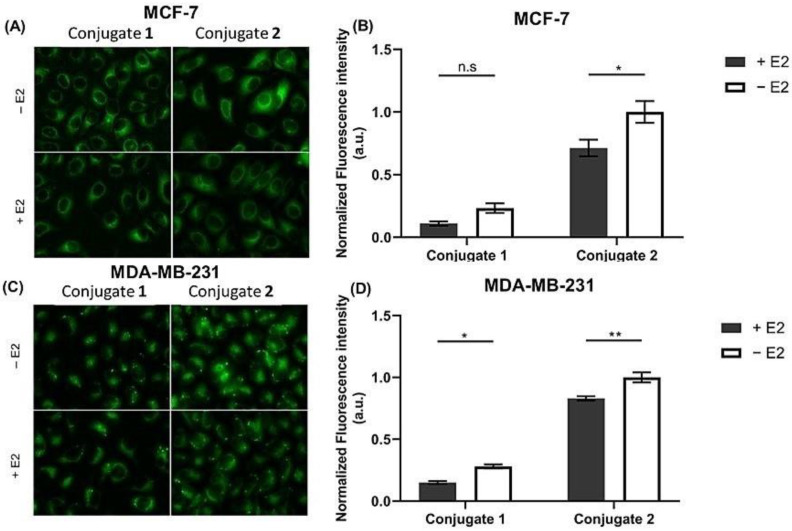
Internalization of estradiol-BODIPY conjugates **1** and **2** in MCF-7 and MDA-MB-231 breast cancer cells with or without E2. MCF-7 and MDA-MB-231 cells were incubated for 1 h with a specific inhibitor (+E2) or without an inhibitor (−E2). After 6 h of incubation with conjugate **1** or **2**, (**A**) MCF-7 and (**C**) MDA-MB-231 cells and the fluorescence intensity were quantified for each cell line, (**B**) and (**D**), respectively. The fluorescence intensity of the cells was corrected for background fluorescence and normalized with vehicle (DMSO). The statistical significance was evaluated by the two-way ANOVA method (* *p* ≤ 0.05, ** *p* ≤ 0.01, n.s—not significant).

**Figure 5:**
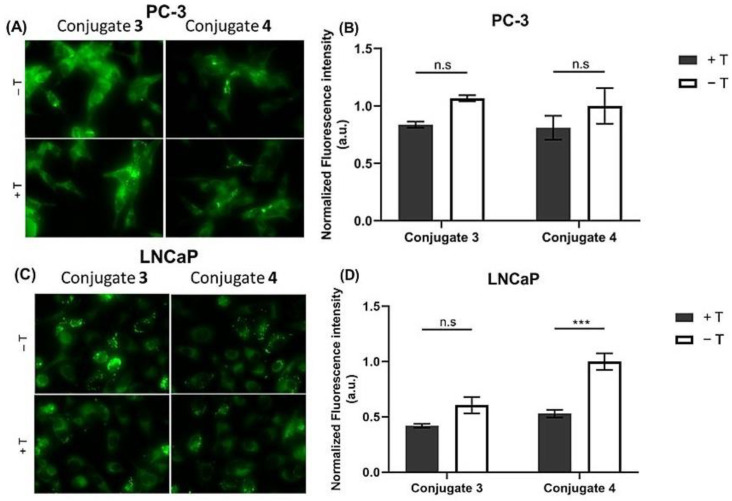
Internalization of 19-nortestosterone-BODIPY conjugates **3** and **4** in PC-3 and LNCaP prostate cancer cells with or without testosterone (T). PC-3 and LNCaP cells were incubated for 1 h with a specific inhibitor (+T) or without an inhibitor (−T). After 6 h of incubation, (**A**) PC-3 and (**B**) LNCaP cells were visualized using an inverted microscope with magnification 400×, and the fluorescence intensity was quantified for each cell line, (**C**,**D**), respectively. The fluorescence intensity of the cells was corrected for background fluorescence and normalized with vehicle (DMSO). The statistical significance was evaluated by the two-way ANOVA method (*** *p* ≤ 0.001, n.s—not significant).

The effects of endocrine disruptors on the steroid-BODIPY conjugates’ internalization were also studied (Figure 4 and Figure 5). The internalization of E2-BODIPY conjugates **1** and **2** in breast cancer cells was evaluated using E2 to block ERα [62]. In the MCF-7 cells, the fluorescence levels of both conjugates slightly decreased after incubation with E2 (Figure 4A,B). Despite their non-responsiveness to ER, we also observed a decrease in E2-BODIPY fluorescence in the MDA-MB-231 cells (Figure 4C,D). In this case, the fluorescence of the BODIPY conjugates was mostly distributed in the cytoplasm.

For the prostate cancer cell lines, a similar experiment was performed using testosterone as a specific ligand for AR. In the AR-non-responsive PC-3 cells, the internalization of both 19-nortestosterone-BODIPY conjugates **3** and **4** decreased when the cells were incubated with testosterone (Figure 5A,B), with the conjugates displaying decreases in their internalization levels of approximately 25% and 20%, respectively (Figure 5B). In the AR-responsive LNCaP cells, similar results were obtained for conjugate **3**, while the internalization levels of conjugate **4** were significantly lower after cell treatment with testosterone, decreasing by approximately 46% (Figure 5C,D). These results suggest that conjugate **4**’s internalization in an AR-responsive cell type is, at least to some extent, dependent on AR responsiveness.

### 2.4. Assessment of Cell Uptake and Trafficking of Steroid-BODIPY Conjugates

#### 2.4.1. Active vs. Passive Transport

To understand the energy dependence of cell uptake in the steroid-BODIPY conjugates, their internalization levels were assessed at two different temperatures, 4 °C and 37 °C. It is known that low temperatures (0–4 °C) can reduce cell metabolism and ATP production, consequently minimizing active transport, for which the optimal temperature is 37 °C [85]. Therefore, if the cellular uptake of a compound occurs at 37 °C but not at 4 °C, it is usually assumed that the transport through the cell membrane is carrier-mediated [85].

**Figure 6:**
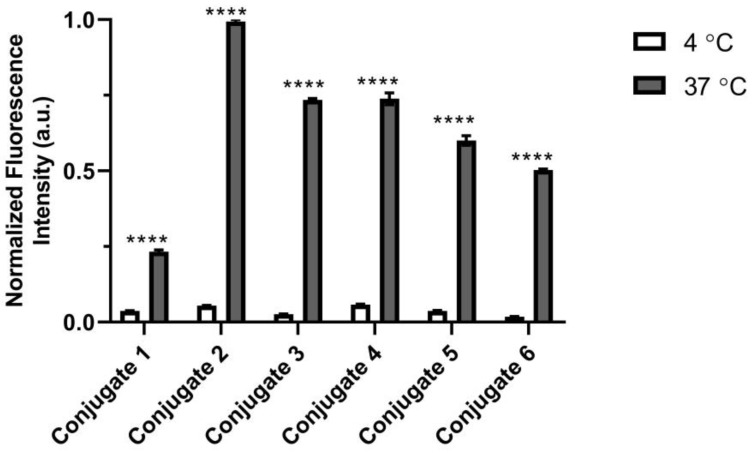
Internalization of steroid-BODIPY conjugates **1**–**6** in PC-3 cells at 4 °C or 37 °C over 6 h. PC-3 cells were seeded for 24 h in complete RPMI medium, and then the medium was replaced with RPMI without phenol red and with 50 μM of estradiol derivates (**1** and **2**) or androgen derivates (**3**–**6**), incubated at 4 °C or 37 °C. After 6 h of incubation, the cells were visualized using an inverted microscope, and images were acquired for the quantification of the fluorescence intensity. The fluorescence intensity of the cells was corrected for background fluorescence and normalized with vehicle (DMSO). Results are expressed as the mean ± SEM of two independent experiments. The statistical significance was evaluated by the two-way ANOVA method (**** *p* ≤ 0.0001).

As shown in Figure 6 (representative fluorescence images in Figure S8), the cellular uptake of all the BODIPY conjugates **1**–**6** by the PC-3 cells, at a low temperature, was significantly lower relative to the uptake levels at 37 °C, which indicates that the internalization of steroid-BODIPY conjugates by these cells is an energy-dependent process.

#### 2.4.2. Inhibition of Endocytosis of Steroid-BODIPY Conjugates

**Figure 7:**
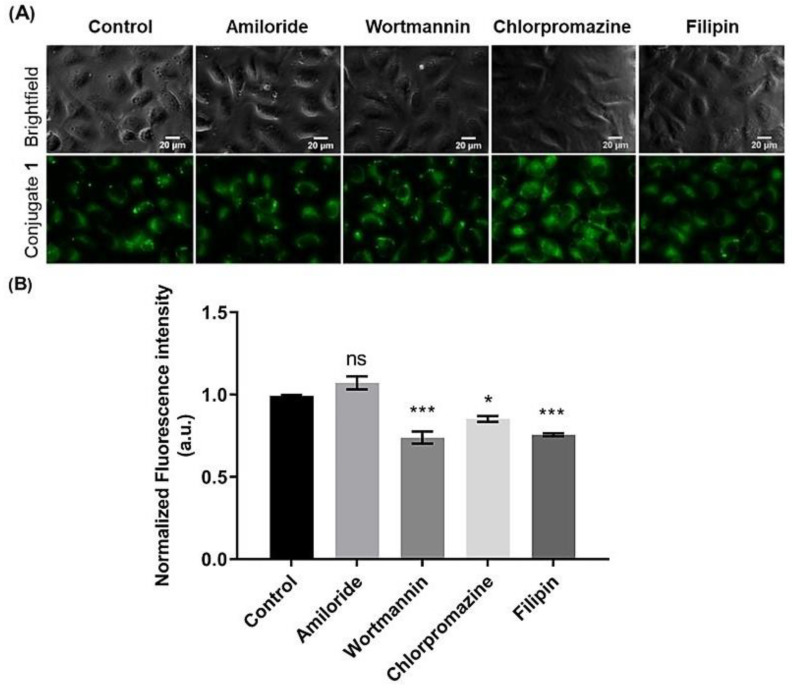
Inhibition of endocytosis of E2-BODIPY conjugate **1** in MDA-MB-231 cells. Cells were incubated for 1 h without endocytosis inhibitor (control) or with four different endocytosis inhibitors—amiloride, wortmannin, chlorpromazine, and filipin. Then, the cells were incubated for 6 h with 0.5% (*v*/*v*) DMSO or 50 μM of conjugate 1 and visualized with an inverted microscope. Images of the conjugate channel with blue excitation (green) (**A**), and quantification of the fluorescence intensity (**B**). The images were treated using Image J software version 1.53c (National Institutes of Health (NIH), Bethesda, MD, USA). The fluorescence intensity of the cells was corrected for background fluorescence and normalized with vehicle (DMSO). The control fluorescence intensity was set as 1 for normalization. Results are expressed as the mean ± SEM of two independent experiments. The statistical significance was evaluated by the one-way ANOVA method (ns—not significant, * *p* ≤ 0.05, *** *p* ≤ 0.001).

As shown in Figure 6, PC-3 cells internalize steroid-BODIPY conjugates through an energy-dependent mechanism. To gain more insight into this internalization mechanism, using E2-BODIPY conjugate **1** as an example, different pharmacological inhibitors of endocytosis-amiloride (EIPA), chlorpromazine, filipin, and wortmannin [86,87,88] were analyzed (Figure 7) in MDA-MB-231 cells (these cells were used due to their lack of ER and AR receptors).

As an inhibitor of NA+/H+ exchange at the cell membrane, amiloride blocks the macropinocytosis pathway [88,89]. The use of this inhibitor had no significant impact on the internalization of conjugate **1** in the TNBC cells (Figure 7B). Conversely, the use of wortmannin, another macropinocytosis inhibitor, resulted in a decrease in cellular uptake when compared to the control or amiloride inhibitor (Figure 7B). Wortmannin is widely used in trafficking studies and has a different mechanism of blocking the endocytic pathway than amiloride, since it is a covalent inhibitor of phosphoinositide 3-kinases (PI3Ks), which are involved in multiple stages of macropinocytosis [90,91]. These results indicate that the internalization of the conjugate is independent of macropinocytosis but requires PI3Ks in a similar way to the process described by Zhao et al., 2019 [92].

Interestingly, a decrease in the green fluorescence intensity was also observed upon pre-treatment with both clathrin- and caveolae-mediated endocytosis inhibitors, chlorpromazine and filipin, respectively (Figure 7). Chlorpromazine is a cationic amphipathic drug that interacts with clathrin from the coated pits, which causes assembly on the endosomal membranes, causing clathrin-mediated endocytosis inhibition [89]. On the other hand, filipin is used to bind cell surface cholesterol and rapidly inhibit caveolae-mediated endocytosis [89]. Together, these results confirm that endocytosis is one of the mechanisms involved in the internalization of the BODIPY-conjugates, with this process being clathrin- and caveolae-mediated and PI3K-dependent.

#### 2.4.3. Intracellular Localization of Steroid-BODIPY Conjugates

**Figure 8:**
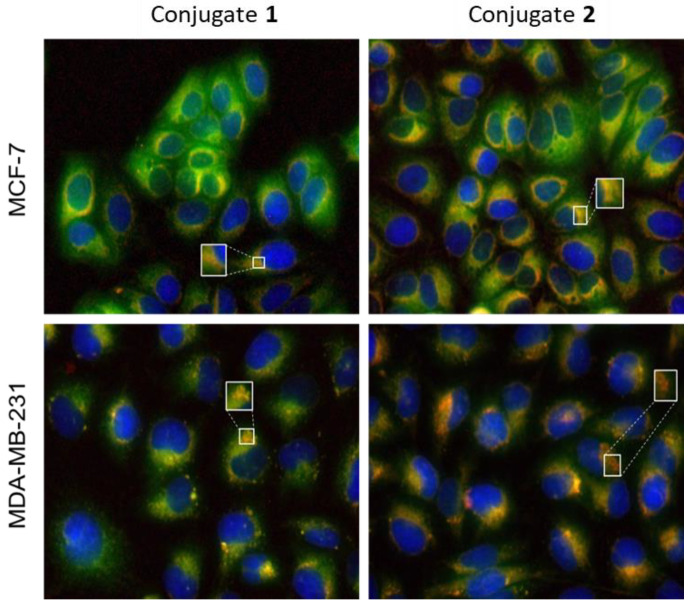
Co-localization of estradiol-BODIPY conjugates **1** and **2** in MCF-7 and MDA-MB-231 breast cancer cells after 6 h of incubation. Cells were stained with Hoechst 33258 (nucleus marker) and LAMP-2 (lysosome marker), and images were acquired with an inverted microscope at magnification 400×. Images were treated using Image J software version 1.53c (National Institutes of Health (NIH), Bethesda, MD, USA). The images are the merged results of images acquired in the different channels (Figure S9). White boxes are the amplification of intracellular regions.

To understand the mechanism of intracellular localization of the steroid-BODIPYs after uptake, cancer cells were stained with a nucleus-selective dye (Hoechst 33258, blue) and a lysosome-selective dye (LAMP-2, red). The results suggest that the cellular distribution of estradiol-BODIPY conjugates **1** and **2** was not spatially uniform throughout the cell (Figure 8 and Figure S9). In fact, it can be observed that the conjugates were spread throughout the cytoplasm, with a clear accumulation around the nuclear membrane (Figure 8). The overlap of the fluorescence of the conjugates (green) with the fluorescence of the lysosome-associated membrane glycoprotein (LAMP-2) (red) suggests that conjugates **1** and **2** were aggregated in the lysosomes. In contrast to the MCF-7 cells, the MDA-MB-231 cells showed greater accumulation in the lysosomal vesicles, suggested by a higher formation of aggregates (Figure 8 and Figure S9). Additionally, differences in co-localization between conjugate **1** and its 11β-methoxy derivate conjugate **2** were also noted, with the latter exhibiting a marked co-localization in both breast cancer cells (Figure 8 and Figure S9).

**Figure 9:**
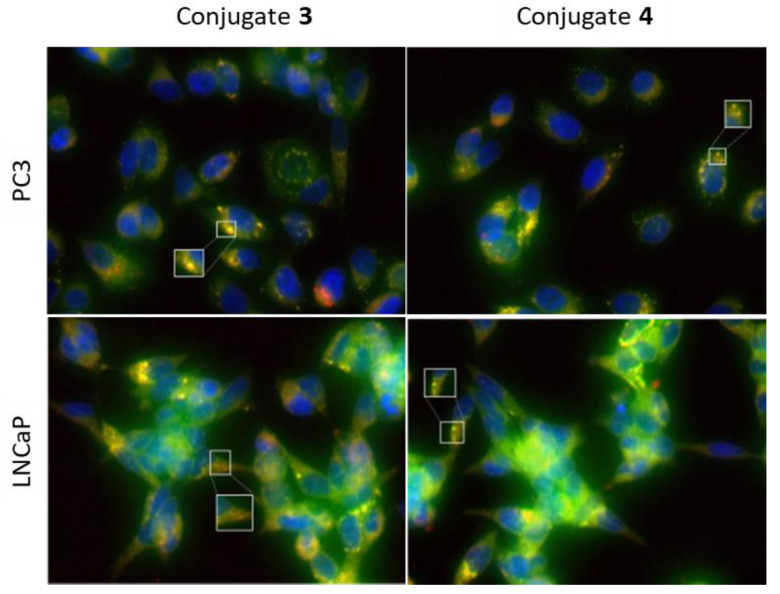
Co-localization of androgen-BODIPY conjugates **3** and **4** in PC-3 and LNCaP prostate cancer cell lines after 6 h of incubation. Cells were stained with Hoechst 33258 (nucleus marker) and LAMP-2 (lysosome marker), and images were acquired with an inverted microscope at 400× magnification. Images were treated using Image J software version 1.53c (National Institutes of Health (NIH), Bethesda, MD, USA). The images are the merged results of images acquired in the different channels (Figure S10). White boxes are the amplification of intracellular regions.

The tracking studies of androgen-BODIPY conjugates **3** and **4** in the PC-3 and LNCaP prostate cancer cells (Figure 9 and Figure S10) demonstrated similar results to those observed for the estradiol conjugates in the breast cancer cells (Figure 8). However, the androgen conjugates’ accumulation around the nuclear membrane occurred to a much lower extent in the prostate LNCaP cancer cells, presenting a more irregular cytosolic distribution (Figure 9 and Figure S10). In the AR-independent prostate cancer PC-3 cells, a clear co-localization of androgen-BODIPY-conjugate- and LAMP-2-derived fluorescence signals was evident, indicating a lysosomal accumulation of these conjugates (Figure 9 and Figure S10).

### 2.5. Visible Light Irradiation

**Figure 10:**
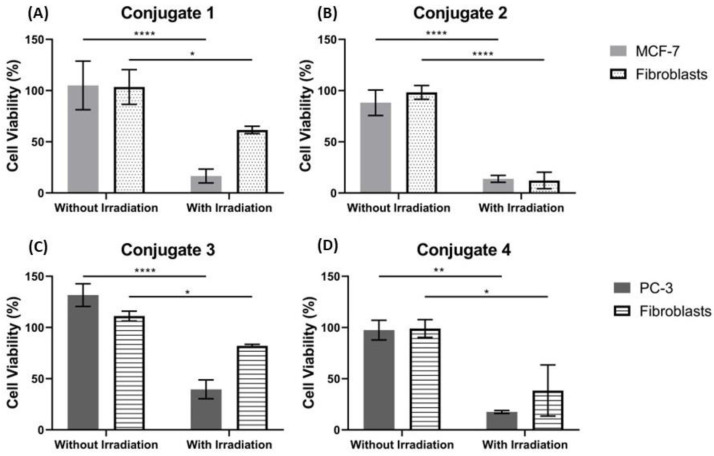
Viability of breast cancer cells MCF-7 and normal dermal fibroblasts following estradiol-BODIPY treatment and visible light irradiation: (**A**) E2-BODIPY conjugate **1** and (**B**) 11β-OMeE2-BODIPY conjugate **2**. Viability of prostate cancer cells and normal dermal fibroblasts after andro-gen-BODIPY treatment and visible light irradiation: (**C**) 9-nortestosterone-BODIPY conjugate **3** and (**D**) 7α-Me-9-nortestosterone-BODIPY conjugate **4**. Cell viability was assessed with MTS assay in MCF-7 and PC-3 cell lines and fibroblasts 24 h after visible light irradiation. Data are normalized against the control (0.25% *v*/*v* DMSO) and expressed as the mean ± SEM of two independent assays. The statistical significance was evaluated by the two-way ANOVA method (* *p* ≤ 0.05; ** *p* ≤ 0.01; **** *p* ≤ 0.0001).

The conjugation of BODIPY dyes to different ligands, such as steroids, can improve the conjugate properties (e.g., targeting selectivity), potentiating their application as breast and prostate cancer diagnostics and therapeutics [93,94]. To evaluate the PDT potential of conjugates **1**–**4** (conjugates **5** and **6** presented lower fluorescence levels), MCF-7 breast cancer cells and normal fibroblasts were incubated with estradiol-BODIPY conjugates **1** and **2** and irradiated with a green light laser. The cell viability was assessed using the MTS assay. The results show that, while the visible light irradiation of the MCF-7 cells or fibroblasts and their incubation with the conjugates alone did not affect the cell viability (Figures S11 and S12), the combination of both resulted in a decline in the MCF-7 cells’ viability of approximately 80% (Figure 10A,B). For conjugate **1**, the cell viability decreased more in the breast cancer cells than in the normal cells (approximately 40% reduction), while for conjugate **2**, the cell viability in the fibroblasts decreased by 80%.

As observed for the estradiol-BODIPY conjugates in the MCF-7 cells, neither androgen-BODIPY conjugates 3 and 4 nor cell irradiation induced significant cell death in the PC-3 prostate cancer cells and fibroblasts (Figures S11 and S12). However, upon irradiation with visible light, both conjugates caused a severe loss of viability in the PC-3 cells, similar to that observed for the estradiol-BODIPY conjugates in the MCF-7 cells (Figure 10). In the case of the fibroblasts, incubation with androgen-conjugates **3** and **4** followed by irradiation reduced the cell viability levels by 25% and 50%, respectively (Figure 10C,D).

## 3. Discussion

The internalization of steroids by cells can occur via several mechanisms, including passive diffusion (an energy-independent mechanism) or transporter-mediated mechanisms such as facilitated diffusion or active transport through specific receptors (energy-dependent mechanisms) [95,96]. Comparing the internalization of estradiol-BODIPY conjugates **1** and **2** between ER-positive MCF-7 and PC-3 cells, ER-negative MDA-MB-231 and LNCaP cells and the primary dermal fibroblasts, revealed that the internalization levels of conjugate **2** was higher in the PC-3 cells (Figure 1, Figure 2 and Figures S2–S7). In general, conjugate **2** showed higher internalization than conjugate **1** in all the cell lines, with MCF-7 presenting a smaller difference between conjugates **1** and **2** (Figure 1A). This may indicate a higher dependency of conjugate **1**’s internalization on ERα due to a higher affinity for the receptor, as previously demonstrated for unconjugated estradiol derivatives [77]. These observations are in line with previously described results, where it was demonstrated that E2-BODIPY conjugate **1** has the highest RBA for ERα among a diverse range of EE2-BODIPY conjugates, reflecting the presence of a long spacer chain in the 17α-position [62,63]. The addition of an 11β-methoxy group to conjugate **2** increases cellular uptake and suggests its potential for application in in vivo targeting.

Comparing the internalization of androgen-BODIPY conjugates **3**–**6** between the AR-positive MCF-7 and LNCaP, the AR-non-responsive cells MDA-MB-231 and PC-3, and the fibroblasts, we showed that 19-nortestosterone-BODIPY conjugates **3** and **4** exhibit higher internalizations than the corresponding testosterone analogs **5** and **6**. This observation is in line with previous results of studies reporting that 17α-ethynyl-nortestosterone (17α-ENT), the parent compound of the androgen-BODIPY conjugates **3** and **4**, showed a significant binding affinity for AR [64]. However, a high internalization of conjugate **4**, disregarding the presence or absence of the receptor (Figure 1, Figure 2 and Figure S7), suggests a low dependence of this conjugate on the receptor. In contrast, conjugate **4**’s internalization in the AR-responsive LNCaP cells was blocked by incubation with testosterone (Figure 5), which suggests the dependence of AR for conjugate uptake. This may be related to an increased binding affinity of 19-nortestosterone-BODIPY conjugate **4** for AR, as previously demonstrated for the structurally related 17α-ethynyl-nortestosterone [64]. Conjugate **4** differs from conjugate **3** in the introduction of a 7α-methyl group to the 19-nortestosterone moiety of conjugate **3**. The increased cell internalization, possibly due to the augmented binding affinity for AR, renders conjugate **4** a promising candidate for in vivo AR targeting. The higher internalization of conjugate **3** in MCF-7 and LNCaP suggests a strong dependency of AR for cellular uptake (Figure 1 and Figure 2). Overall, our results suggest that the selected estradiol- and androgen-BODIPY conjugates display relative selectivity towards cells expressing their specific receptors. Moreover, the fluorescence signal in the intracellular vesicles suggests that an endocytic pathway is the route of internalization of the tested estradiol-BODIPY conjugates.

The cellular uptake of E2-BODIPY conjugate **1** in the ER-negative MDA-MB-231 cells reveals that internalization occurs through an energy-dependent mechanism (Figure 6) as part of a caveolin- and clathrin-mediated and PI3K-dependent process (Figure 7). Most steroid receptors are situated in the cell nucleus [97]. However, a small proportion (approximately 5%) of different steroid receptors, namely ERs, PR, and AR, are localized at the cell membrane and cytosol [97,98]. In addition, it has been reported that receptors for estrogen, androgen, and progesterone bind at the plasma membrane and in the nuclei of breast and prostate cancer cells, influencing tumor cell biology [97,98]. In previous reports, it was demonstrated that membrane pools of steroid receptors are associated with a caveolin-1 (CAV1) structural coat protein of caveolae [98]. For example, estradiol is highly concentrated in isolated caveolae, immediately inducing ERα binding to caveolin-1, which serves as a scaffold for membrane-localized signaling molecules [98]. Although lacking ER expression, caveolae-mediated endocytosis is a possible endocytic pathway for the cellular uptake of the studied conjugates in both ER− and ER+ cell lines. This hypothesis was substantiated by the co-localization of the steroid-BODIPY conjugates with lysosomes (Figure 8 and Figure 9). In the endocytosis process, an invagination of the cell membrane, along with the cell surface receptors and soluble molecules, occurs, forming endosomes [99]. Lysosomes constitute the final stage of the degradative endocytic pathway [99]. Lysosome-associated membrane glycoprotein 2 (LAMP-2), which is located at the lysosomal membrane, maintains lysosomal stability, participates in autophagy, and is crucial for lysosomal function [100,101,102]. These cellular compartments digest and recycle extracellular and intracellular materials [100,101,102]. Evidence suggests that steroid receptors can exist in different subcellular locations that, therefore, are required for the complete action of steroid hormones [98]. Our results suggest that the steroid-BODIPY conjugates can be internalized through caveolae-mediated endocytic routes (Figure 7) and shuttled to the lysosomes (Figure 8 and Figure 9).

In general, we observed that all the conjugates are non-toxic to cancer and normal cells (Figure S11) and present higher selectivity towards cancer cells in a 2D co-culture of cancer cells and normal dermal fibroblasts (Figure 3), making them suitable for certain cancer therapies, such as PDT. PDT is a combined therapeutic method for cancer treatment that possesses three important elements for its effective activity: a photosensitizer, light irradiation, and oxygen [103,104,105]. None of these components, individually, produces a biological response. However, a mixture of the three components promotes the formation of reactive oxygen species (ROS), triggering irreversible damage [103,104,105]. Our results indicate that these steroid-BODIPY conjugates, particularly conjugates **1** and **3**, are potential candidate photosensitizers for PDT against breast and prostate cancer, respectively (Figure 10).

## 4. Materials and Methods

### 4.1. Steroid-BODIPY Conjugates

All steroid-BODIPY conjugates were prepared and characterized as previously detailed (Scheme 1 and Scheme 2) [62,63]. The analytical pure samples (>98%) used for the cell-based studies were obtained by semi-preparative HPLC on C-18 reversed-phase or silica gel columns [63]. Briefly, their preparation involved the attachment of an iodo-derivative of BODIPY to the C17α-ethynyl or -heptynyl substituent of the steroid precursor using the Sonogashira coupling reaction conditions. The following steroid-BODIPY conjugates were used in these studies: conjugate **1** (17α-[(4-ethynylphenyl)-(4,4-difluoro-8-(1,3,5,7-tetramethyl-4-bora-3a,4a-diaza-s-indacene)]-3,17β-estradiol) and conjugate **2** (17α-[(4-ethynylphenyl)-(4,4-difluoro-8-(1,3,5,7-tetramethyl-4-bora-3a,4a-diaza-s-indacene)]-11β-OMe-3,17β-estradiol), and conjugate **3** (17α-[4,4-difluoro-8-(4-ethynylphenyl)-1,3,5,7-tetramethyl-4-bora-3a,4a-diaza-s-indacene]-19-nortestosterone), conjugate **4** (17α-[4,4-difluoro-8-(4-ethynylphenyl)-1,3,5,7-tetramethyl-4-bora-3a,4a-diaza-s-indacene]-7α-Me-19-nortestosterone), conjugate **5** (17α-[4,4-difluoro-8-(4-ethynylphenyl)-1,3,5,7-tetramethyl-4-bora-3a,4a-diaza-s-indacene]-testosterone), and conjugate **6** (17α-[4,4-difluoro-8-(4-ethynylphenyl)-1,3,5,7-tetramethyl-4-bora-3a,4a-diaza-s-indacene]-7α-Me-testosterone). All conjugates showed similar absorption profiles: UV − Vis (CH_2_Cl_2_) λ_max_/nm (log ε): 500 (4.99).

### 4.2. Cell Culture

Two human-prostate-carcinoma-derived cancer cell lines (PC-3 and LNCaP) were cultivated in Roswell Park Memorial Institute (RPMI) 1640 culture medium (ThermoFisher Scientific, Waltham, MA, USA), and two human-breast-adenocarcinoma-derived cancer cell lines (MCF-7 and MDA-MB-231) and normal human dermal fibroblasts were cultured in Dulbecco’s modified Eagle’s medium (DMEM, ThermoFisher Scientific). All culture media were supplemented with 10% (*v*/*v*) fetal bovine serum (FBS, ThermoFisher Scientific) and a mix solution of 100 U/mL penicillin and 100 µg/mL streptomycin (ThermoFisher Scientific). Additionally, the MCF-7 and MDA-MB-231 culture media were also supplemented with 1% (*v*/*v*) MEM, a non-essential amino acid (ThermoFisher Scientific). The cell cultures were maintained at 37 °C in a humidified atmosphere of 5% (*v*/*v*) CO_2_. All cell lines were purchased from the American Type Culture Collection (ATCC, Manassas, VA, USA). The cell culture characteristics are summarized in Table 1. For more details, please consult Figure S1.

### 4.3. Cellular Internalization of Steroid-BODIPY Conjugates

The BODIPY fluorescence properties were investigated by fluorescence microscopy with a Ti-U Eclipse inverted microscope (Nikon, Tokyo, Japan) equipped with DAPI (excitation at 360/40 nm and emission at 460/50 nm), FITC (excitation at 480/30 nm and emission at 535/40 nm), and G2A (excitation at 535/50 nm and emission > 590 nm) fluorescence filters (Nikon). The intensity of the fluorescence in each image was analyzed with Image J software version 1.53c (National Institutes of Health (NIH), Bethesda, MD, USA) [106] by quantifying the corrected total cell fluorescence (CTCF) = integrated density—(area of cell × background mean fluorescence) for each cell and by normalizing it against the respective non-treated control sample at 0 h [107]. The CTCFs were quantified for at least five cells per image, acquired in more than three random microscopic fields in triplicate for each cell line. All fluorescence studies were performed after replacing the cell culture medium with non-supplemented medium without phenol red (ThermoFisher Scientific).

#### 4.3.1. Internalization in 2D Monocultures

The cell lines were seeded at a density of 1 × 10^5^ cells per well on a 24-well plate and incubated for 24 h for adhesion (2D monolayers). After the medium in each well was replaced with fresh media (without phenol red) supplemented with 50 μM of each conjugate or 0.5% (*v*/*v*) DMSO (vector control), the cells were imaged after 0 h, 45 min, 2 h, and 6 h of incubation with a FITC filter and with brightfield.

#### 4.3.2. Internalization in 2D Co-Cultures

MDA-MB-231 or PC-3 cells were seeded with fibroblasts in proportions of 1:1 (2D co-cultures). After 24 h, the medium was replaced with fresh medium (without phenol red) supplemented with 5 μM or 25 μM of each conjugate or 0.05% (*v*/*v*) and 0.25% (*v*/*v*) DMSO (vector control), respectively. The cells were visualized at 0 h and 6 h with a FITC filter and with brightfield.

#### 4.3.3. Effects of Endocrine Disruptors on the Internalization of BODIPY-Conjugates in 2D Monolayers

All cell lines were seeded on 24-well plates at a cell density of 1 × 10^5^ cells per well. After 24 h, the culture medium was replaced with DMEM/F-12 (Nutrient Mixture F-12) medium (ThermoFisher Scientific) supplemented with hormone-depleted FBS (charcoal-stripped FBS, ThermoFisher Scientific), hereafter referred to as hormone-depleted medium, or DMEM/F-12 medium supplemented with regular FBS, hereafter referred to as normal medium. After overnight incubation, the medium was replaced with fresh DMEM/F-12 (with or without hormones) supplemented with 10 μM testosterone inhibitor (Sigma Aldrich, St. Louis, MO, USA) or 17α-ethynylestradiol (EE2) (Sigma Aldrich). After 1 h, the medium was replaced with fresh hormone-depleted or normal DMEM/F-12 medium supplemented with 25 μM BODIPY conjugate or 0.25% (*v*/*v*) DMSO. The cells were imaged after 0 h and 6 h with a FITC filter and with brightfield.

#### 4.3.4. Active vs. Passive Transport

PC-3 cells were seeded at 1 × 10^5^ cells/well and incubated for 24 h. Afterwards, the culture medium was replaced with fresh medium supplemented with 50 μM of each BODIPY conjugate or 0.5% (*v*/*v*) DMSO (vehicle control) and incubated at 37 °C or 4 °C. After 6 h of incubation, the cells were imaged with a FITC filter and with brightfield.

#### 4.3.5. Evaluation of Endocytosis Internalization

MDA-MB-231 cells were seeded at 1 × 10^5^ cells/well on a 24-well plate. After 24 h, the cells were treated with the following endocytic inhibitors for 2 h: 5 μM filipin III (Sigma Aldrich), 30 μM chlorpromazine hydrochloride (Sigma Aldrich), 12.5 μM amiloride hydrochloride (Sigma Aldrich), or 0.3 μM wortmannin (Sigma Aldrich). The culture medium was replaced with fresh culture medium supplemented with conjugate **1**. The cell fluorescence was quantified after 6 h of incubation with a FITC filter and with brightfield.

#### 4.3.6. Intracellular Tracking

To understand the intracellular localization of steroid-BODIPY conjugates in cancer cells, a fluorescence microscopy assay was performed, where the nucleus and lysosomes were stained using Hoechst 33258 dye (Phenol, 4-[5-(4-methyl-1-piperazinyl) [2,5′-bi-1H-benzimidazol]-2′-yl]-, trihydrochloride 23491-45-4) (ThermoFisher Scientific) and anti-LAMP-2A antibody (Abcam, Cambridge, UK), respectively. The cells were seeded on 24-well plates at a density of 1 × 10^5^ cells/well and incubated as described in Section 4.3.1. After 6 h, the cells were fixed with 4% (*w*/*v*) formaldehyde for 20 min at room temperature (RT) and permeabilized with 0.1% (*v*/*v*) Triton-X 100 for 5 min. The cells were incubated with 7.5 μg/mL Hoechst 33258 for 15 min at RT, washed three times with PBS, and then incubated with 1% bovine serum albumin (BSA) for 30 min at RT. Afterwards, the cells were incubated with anti-LAMP2A antibody (1:200) (ab125068, Abcam) for 1 h at RT and for 30 min at RT with the respective secondary antibody, goat anti-rabbit IgG (TRITC) (1:2000) (ab6718; Abcam). Three different images were acquired for the same microscopic frame. Images of the nucleus were acquired with the DAPI filter, images of LAMP-2 were acquired with the G2A filter, and images of the steroid-BODIPY conjugates were acquired with the FITC filter.

### 4.4. Cell Viability

The normal dermal fibroblasts and cancer cell lines MCF-7, MDA-MB-231, PC-3, and LNCaP were seeded at 0.75 × 10^4^/well on a 96-well plate. After 24 h, the medium was replaced with fresh media supplemented with 50 μM of BODIPY conjugate, 0.5% (*v*/*v*) DMSO, or 0.4 μM of doxorubicin (positive control). The cells were then incubated for 6 h and 24 h, and the cell viability was determined using the Cell Titer 96^®^ Aqueous One solution cell proliferation assay (Promega, Madison, WI, USA). In this method, an inner salt, 3-(4,5-dimethylthiazol-2-yl)-5-(3-carboxymethoxyphenyl)-2-(4-sulfophenyl)-2H-tetrazolium (MTS), is bio-reduced into a colored formazan product that is soluble in culture medium. This conversion is achieved using mitochondrial dehydrogenases (NADPH or NADH) present in metabolically active cells in the presence of the phenazine methosulfate (PMS) that is used as an electron coupling reagent. Formazan production, measured by absorbance at 490 nm, is directly proportional to the number of living cells in the culture medium [108,109].

### 4.5. Visible-Light Irradiation

The normal dermal fibroblasts and cancer cells MCF-7 and PC-3 were seeded at a density of 0.75 × 10^4^ cells/well on a 96-well plate. After 24 h, the medium was replaced with fresh medium supplemented with 25 μM BODIPY conjugate, 0.25% (*v*/*v*) DMSO, or 0.4 μM doxorubicin. After 6 h, the cells were irradiated with green laser light for 60 s using a continuous 532 nm green-diode-pumped solid-state laser (Changchun New Industries Optoelectronics Tech. Co., Ltd., Changchun, China) coupled with an optical fiber, with a laser diode intensity (LDI) of 2.45 W/cm^2^ (see Figure S13). Afterwards, the cells were incubated for 24 h, and their viability was assessed as described in Section 2.4.

### 4.6. Statistical Analysis

All results are presented as the mean ± SEM of two independent biological assays, each obtained by technical duplicates. The statistical analysis of data was performed using the GraphPad Prism program 6 (GraphPad Software, La Jolla, CA, USA). The ANOVA tests with multiple comparisons were performed to compare the different experimental groups of data for statistical significance. Results with *p* < 0.05 were considered statistically significant.

## 5. Conclusions

In this work, we reported the potential theranostic properties (imaging and PDT) of estradiol, 19-nortestosterone, and testosterone derivatives conjugated to BODIPY. The cellular uptake and trafficking of the conjugates were studied in breast and prostate cancer cells expressing different combinations (or absence) of ER and AR using fluorescence microscopy. A low level of specificity for the internalization of steroid-BODIPY conjugates in the different cancer cell lines was observed. The highest internalization was observed in 11β-OMe-estradiol-BODIPY **2** and 7α-Me-19-nortestosterone-BODIPY **4** towards cells expressing their specific receptors. Blocking experiments showed changes in non-specific cell uptake in the cancer and normal cells, which likely reflect differences in the lipophilicity of the conjugates. In the co-cultures of breast or prostate cancer cells with normal fibroblasts, when used in higher concentrations, the steroid-BODIPY conjugates were internalized with higher specificity in cancer cells as compared to fibroblasts (in which internalization was low or absent), demonstrating their suitability for the targeting of cancer cells within the tumor microenvironment.

The steroid-BODIPY conjugates’ internalization pathways were assessed by fluorescence microscopy, demonstrating a significant decrease in their uptake at low temperatures, indicating that these conjugates enter cancer cells through an energy-dependent process. To verify whether this internalization is dependent on endocytic pathways, the effects of different pharmacologic inhibitors of endocytosis were monitored. We conclude that the internalization of the steroid-BODIPY conjugates occurs through clathrin- and caveolae-mediated endocytosis in a PI3K-dependent mechanism. This hypothesis for the endocytic internalization of these conjugates was also corroborated by the co-localization of the steroid-BODIPY conjugates and lysosomes.

The cell viability assays demonstrated the strong antiproliferative activity of the steroid-BODIPY conjugates in combination with visible light irradiation, suggesting their potential use as photosensitizers, especially in the case of estradiol conjugates **1** and 19-nortestosterone conjugate **3** in breast- and prostate-cancer-targeted PDT, respectively.

## Figures and Tables

**Table 1:** Origin and receptor (AR, ER+, ER−) status of the cell cultures.

Cell Line	ATCC Reference	Origin	AR	ER	Reference
MCF-7	HTB-22	Metastatic breast adenocarcinoma	+	+	[66,68,81]
MDA-MB-231	CRM-HTB-26	Metastatic breast adenocarcinoma	−	−	[66,69,81]
PC-3	CRL-1435	Metastatic prostate adenocarcinoma; Grade IV	−	+	[70,71]
LNCaP	CRL-1740	Metastatic prostate carcinoma	+	−	[72]
Fibroblasts	PCS-201-010	Primary normal dermal, neonatal	−	−	This work (Figure S1)

## Data Availability

The data presented in this study are available in the article.

## Supplementary Materials

The following supporting information can be downloaded at: https://www.mdpi.com/article/10.3390/ijms24043600/s1.
