# Supplementary material for: Cell Uptake of Steroid-BODIPY Conjugates and Their Internalization Mechanisms: Cancer Theranostic Dyes
Source: Int J Mol Sci. 2023 Feb 10;24(4):3600. doi: 10.3390/ijms24043600 (PMC9963437; doi:10.3390/ijms24043600)
Supplement: Supplementary file 1 [file ijms-24-03600-s001.zip › ijms-2181537-supplementary.docx]

**Supplementary information**

**Cell uptake of steroid-BODIPY conjugates and their internalization mechanisms: Cancer theranostic dyes**

Ana F. Amendoeira ^1, 2, †^, André Luz ^1,2, †^, Ruben Valente ^1,2^, Catarina Roma-Rodrigues ^1,2^, Hasrat Ali ^3^, Johan E. van Lier ^3^, Fernanda Marques ^4^, Pedro V. Baptista ^1,2, *^ and Alexandra R. Fernandes ^1,2, *^

^1^ Associate Laboratory i4HB, Institute for Health and Bioeconomy, NOVA School of Science and Technology, 2819-516 Caparica, Portugal

^2^ UCIBIO—Applied Molecular Biosciences Unit, Department of Life Sciences, NOVA School of Science and Technology, 2819-516 Caparica, Portugal

^3^ Department of Nuclear Medicine and Radiobiology, Faculty of Medicine and Health Sciences, University of Sherbrooke, Sherbrooke, QC J1H5N4 Canada

^4^ Centro de Ciências e Tecnologias Nucleares, Departamento de Engenharia e Ciências Nucleares, Instituto Superior Técnico, Universidade de Lisboa, Estrada Nacional 10, km 139.7, 2695-066 Bobadela, Portugal

* Correspondence: pmvb@fct.unl.pt (P.V.B.); ma.fernandes@fct.unl.pt (A.R.F.)

† These authors contributed equally to this work.

**Expression of steroid receptors in Fibroblasts by Western-blot**


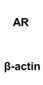

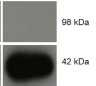

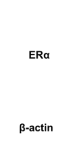

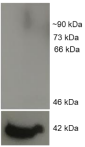


**Figure S1.** Representative images of western blot results of Estrogen α Receptor (ERα) and Androgen Receptor (AR) expression in Fibroblasts. 50 ng of protein extracted from fibroblasts were separated by a 10% acrylamide-bisacrylamide gel (SDS-PAGE) and then transferred to PVDF membranes (Amersham^TM^ Hybond^TM^, Life Sciences) using a semi-dry system transfer (. After blocking with a 5 % (w/v) non-fat milk solution in TBST (50 mM Tris, 150 mM NaCl and 0.1% (v/v) Tween 20, pH 7.5) during 1 h at RT with agitation, the membranes were incubated with a primary antibody in 5% non-fat milk in TBST, anti-androgen receptor (1:5000) (Abcam) or anti-estrogen receptor α (1:500) (Sigma Aldrich), for 1h at RT with agitation or overnight at 4 °C, respectively. After primary antibody incubation, the membrane was washed TBST 1x and incubated with the appropriate secondary antibody, anti-rabbit IgG HPR-linked antibody (1:2000) (Cell Signalling Technology) for 1 h with agitation. In order to detect protein bands (AR have a molecular weight of 98KDa and Erα could have three isoforms: full-length ERα with a molecular weight of 66 kDa and the two truncated isoforms, ERα36 and ERα46 with molecular weights of 36 and 46 kDa in the membrane) a WesternBright ECL substrate (Advansta) as applied to the membrane and incubated for 5 min. After ECL incubation, the film was exposed to the membrane in light absence. β-actin was used as control. The same procedure of blocking and antibodies incubation described previously was done with anti-β-actin (1:5000) (Sigma Aldrich) primary antibody, and anti-mouse IgG HPR-linked antibody (1:3000) (Sigma Aldrich) as secondary antibody.

**Internalization and Intracellular tracking of steroid-BODIPY conjugates in cancer and normal cells**


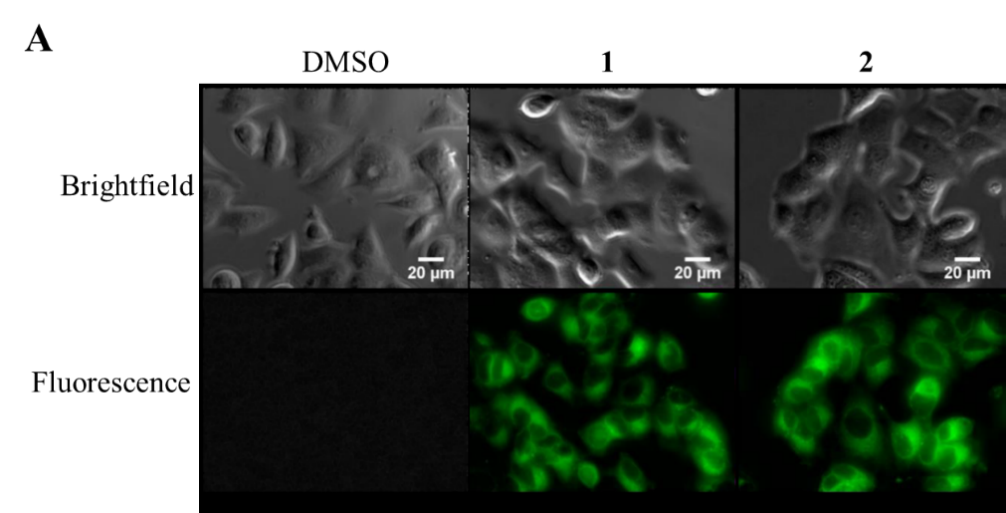

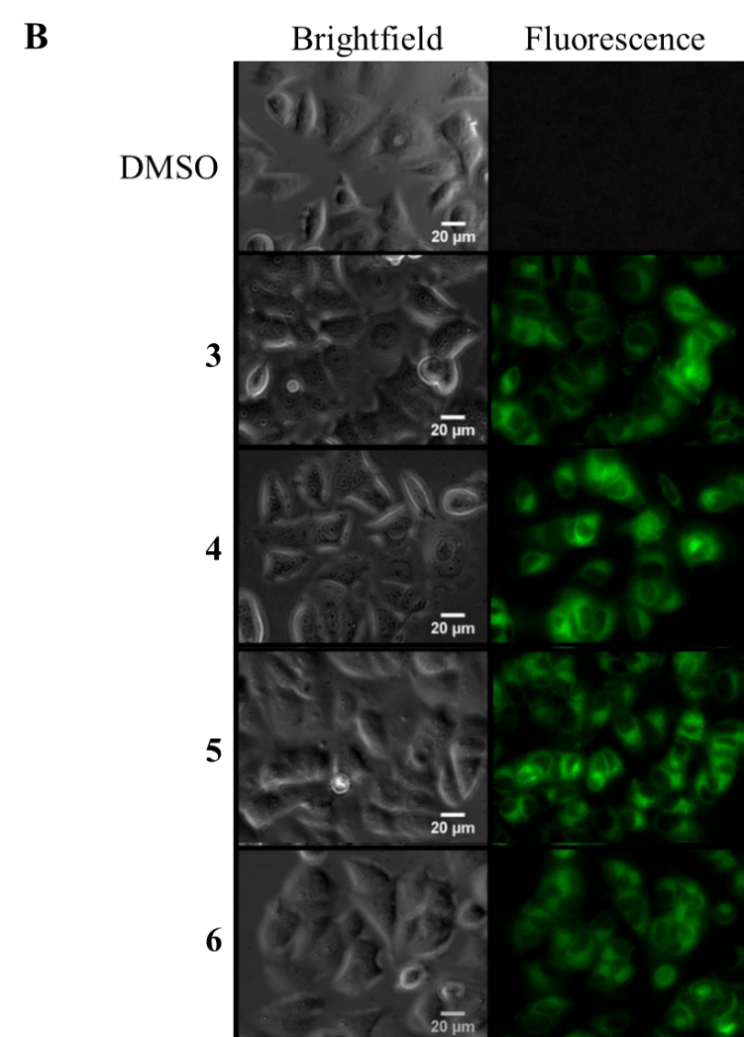


**Figure S2.** Brightfield and fluorescence images of (A) estrogen-BODIPY conjugates **1-2** and (B) androgen-BODIPY conjugates **3-6** in MCF-7 breast cancer cell line after 6h incubation. MCF-7 cells were seeded for 24h in complete DMEM medium (with Phenol red) supplemented with MEM and then medium was replaced with DMEM without phenol red and 0.5% (v/v) DMSO (control) or 50 μM of conjugates **1-6**.

**
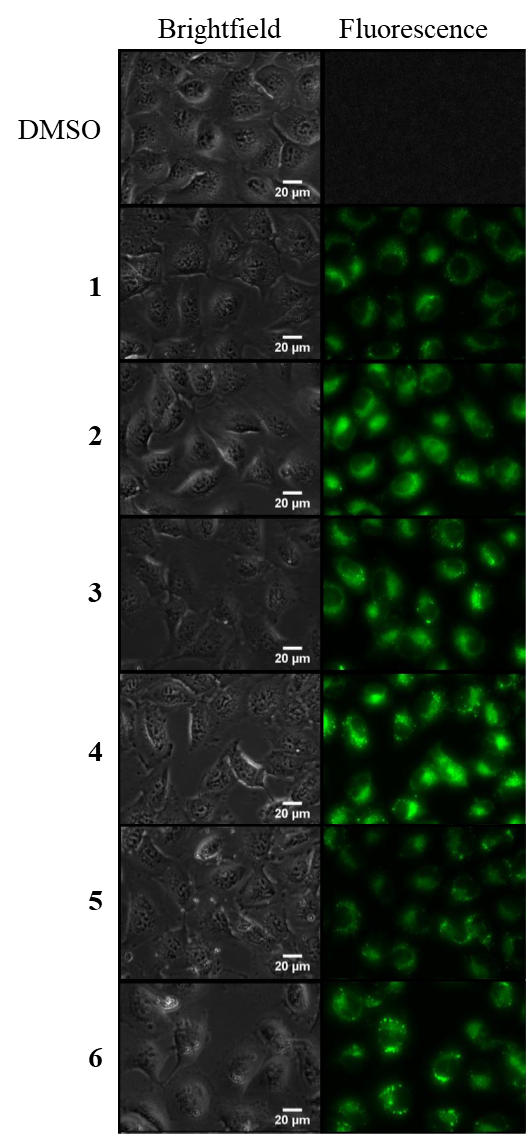
**

**Figure S3.** Brightfield and fluorescence images of estrogen-BODIPY conjugates **1-2** and androgen-BODIPY conjugates **3-6** in MDA-MB-231 breast cancer cell line after 6h incubation. MDA-MB-231 cells were seeded for 24h in complete DMEM medium (with Phenol red) supplemented with MEM and then medium was replaced with DMEM without phenol red and 0.5% (v/v) DMSO (control) or 50 μM of conjugates **1-6**.


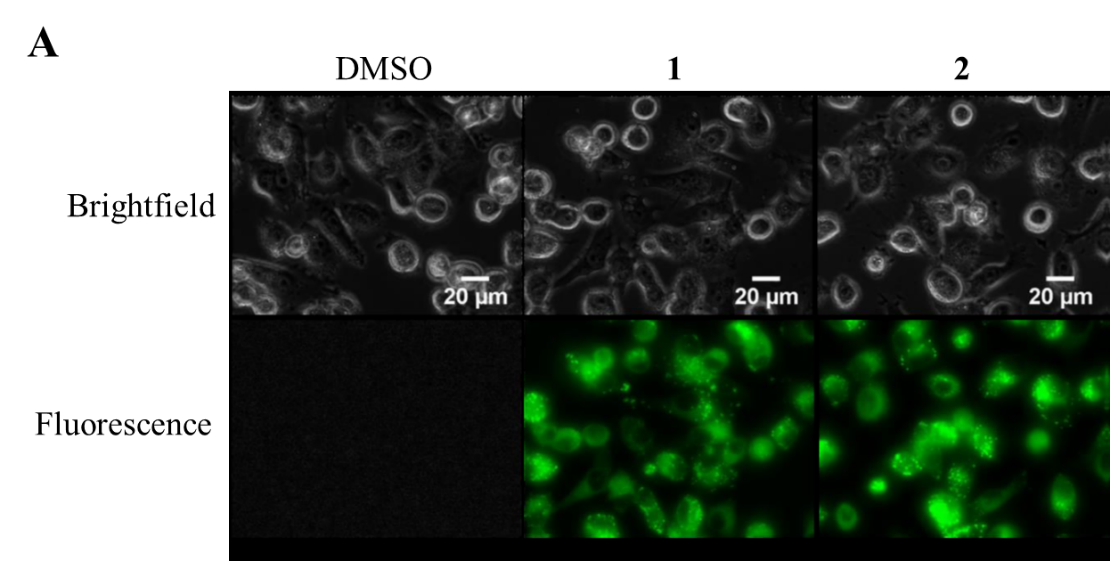

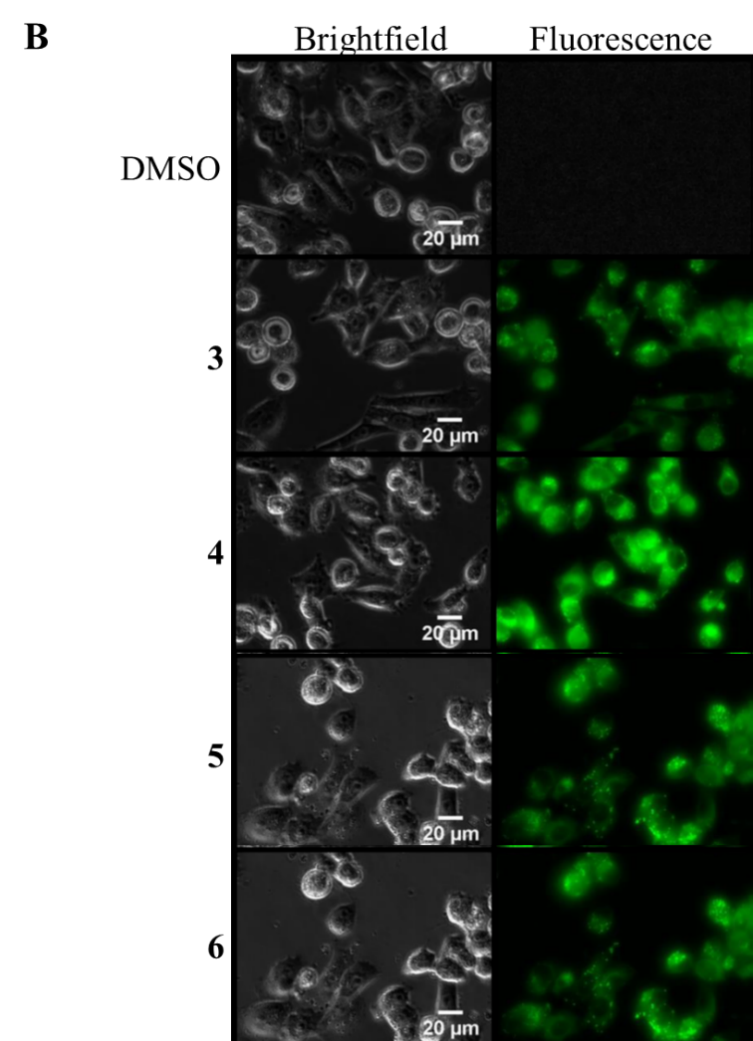


**Figure S4.** Brightfield and fluorescence images of (A) estrogen-BODIPY conjugates **1-2** and (B) androgen-BODIPY conjugates **3-6** in PC3 prostate cancer cell line after 6h incubation. PC3 cells were seeded for 24h in complete RPMI medium (with Phenol red) and then medium was replaced with RPMI without phenol red, supplemented with 0.5% (v/v) DMSO (control) or 50 μM of conjugates **1-6.**

*
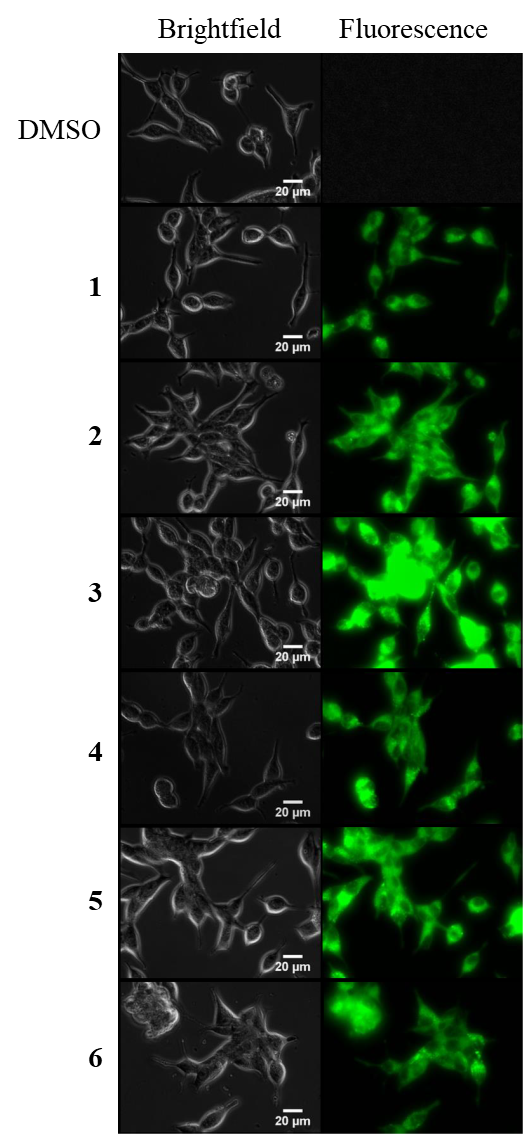
*

**Figure S5.** Brightfield and fluorescence images of estrogen-BODIPY conjugates **1-2** and androgen-BODIPY conjugates **3-6** in LNCaP prostate cancer cell line after 6h incubation. LNCaP cells were seeded for 24h in complete RPMI medium (with Phenol red), when medium was replaced with RPMI without phenol red supplemented with 0.5% (v/v) DMSO (control) or 50 μM of conjugates **1-6**.

*Steroid-BODIPY conjugates in primary normal Fibroblasts*

All the internalization studies in fibroblasts were evaluated after 6 h of incubation with the conjugates (maximum level of fluorescence attained with the other tumor cell models) and the results are shown in **Figure S6** and the normalized fluorescence intensity in **Figure S7**. Except the EE2-C8 and HA-4200 (testosterone derivate), with the lower fluorescence intensity, all conjugates show similar fluorescence levels in fibroblasts (**Figure S6**). Similar to the results in the cancer cell models, green fluorescence is observed for all the BODIPY conjugates in fibroblasts (**Figures S5** and **S6**), that might be explained to some cell permeability to BODIPY conjugates or receptor-mediated independent mechanism, such as endocytosis.

**
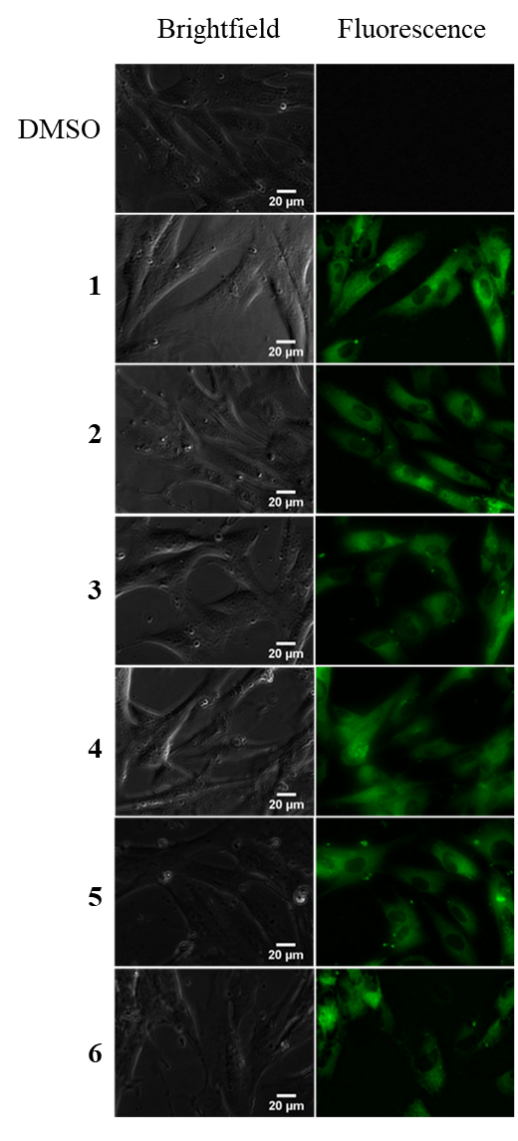
**

**Figure S6.** Brightfield and fluorescence images of estrogen-BODIPY conjugates **1-2** and androgen-BODIPY conjugates **3-6** in normal dermal fibroblasts after 6h incubation. Fibroblasts were seeded in DMEM medium, that after 24h was replaced with DMEM without phenol red supplemented with 0.5% (v/v) DMSO (control) or 50 μM of conjugates **1-6**.

**Figure S7.** Normalized fluorescence intensity of Fibroblasts incubated for 6 h with steroid-BODIPY conjugates. Fluorescence intensity of the cells was corrected for background fluorescence and normalized with vehicle (DMSO). Results are expressed as the mean ± SEM of two independent experiment in duplicate.

**Assessment of cell uptake and trafficking of steroid-BODIPY conjugates**


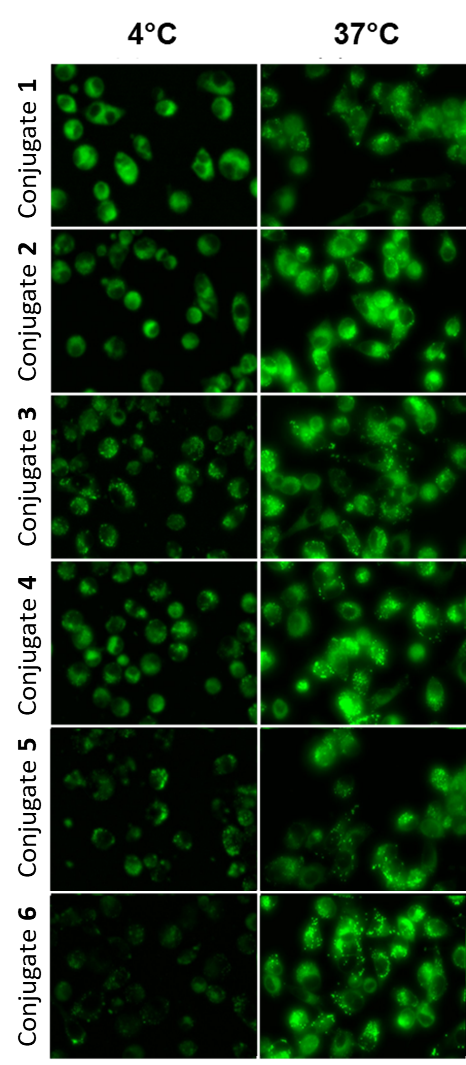


**4 °C**

**37 °C**

**Figure S8.** Fluorescence images of steroid-BODIPY conjugates **1-6** in PC-3 cells. PC-3 cells were seeded for 24h in complete RPMI medium (with Phenol red), the medium was replaced with RPMI without phenol red supplemented with 50 μM of conjugate **1-6**, and cells were incubated at 4 °C or 37 °C for 6h. Magnification 400x.

**
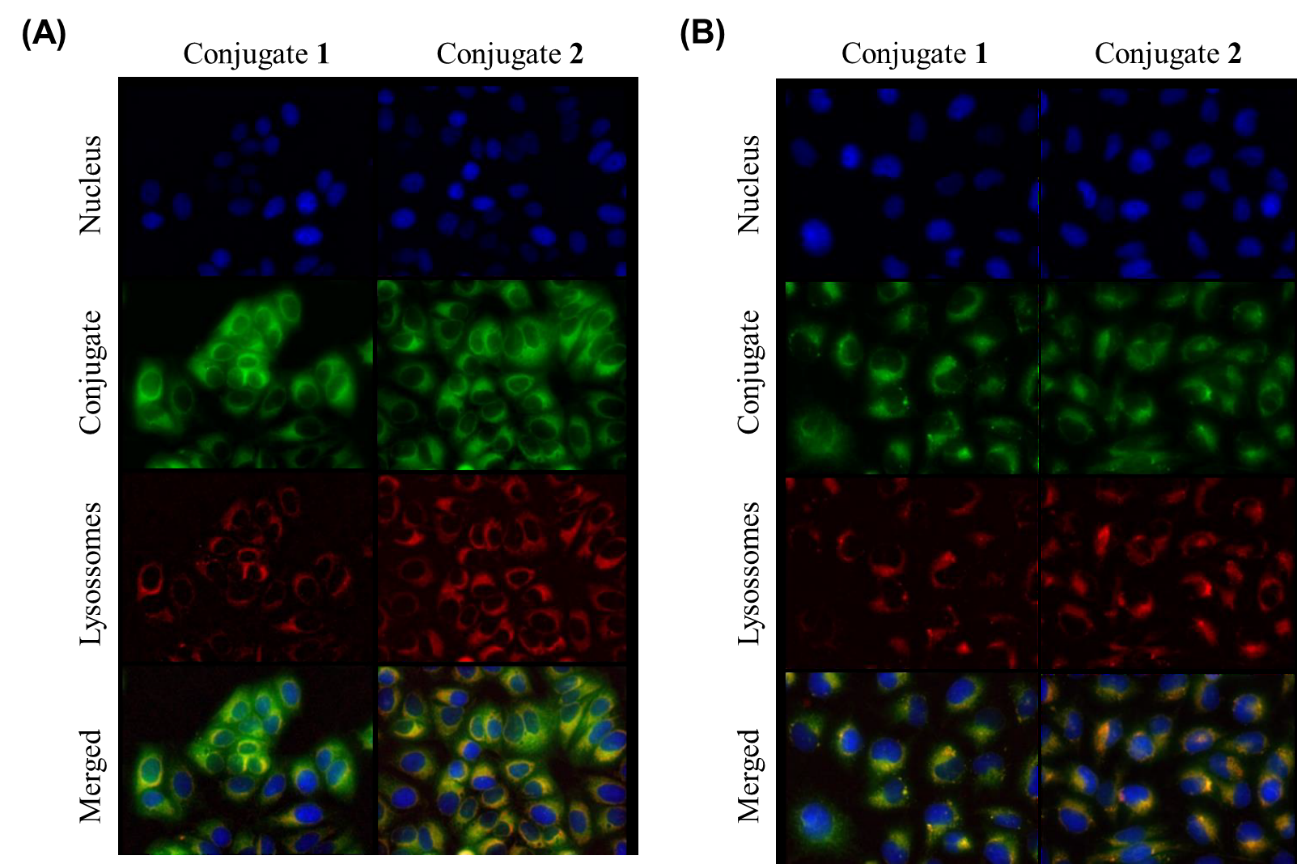
**

**Figure S9.** Intracellular localization of estradiol-BODIPY conjugates **1** and **2** in (A) MCF-7 and (B) MDA-MB-231 breast cancer cell lines after 6h incubation. MCF-7 and MDA-MB-231 cells were seeded for 24h in complete DMEM medium (with Phenol red) supplemented with MEM and then medium was replaced with DMEM without phenol red and 50 μM of conjugates **1** and **2**. After fixation with formaldehyde and permeabilization with Triton X-100, cells were stained with Hoechst 33258 (nucleus marker) and LAMP-2 (lysosome marker). Cells were visualized on inverted microscope with amplification 400x and images of Hoechst channel were acquired with UV excitation (blue), LAMP-2 channel with green excitation (red) and conjugate channel with blue excitation (green). The presented fluorescence images were treated using Image J software.


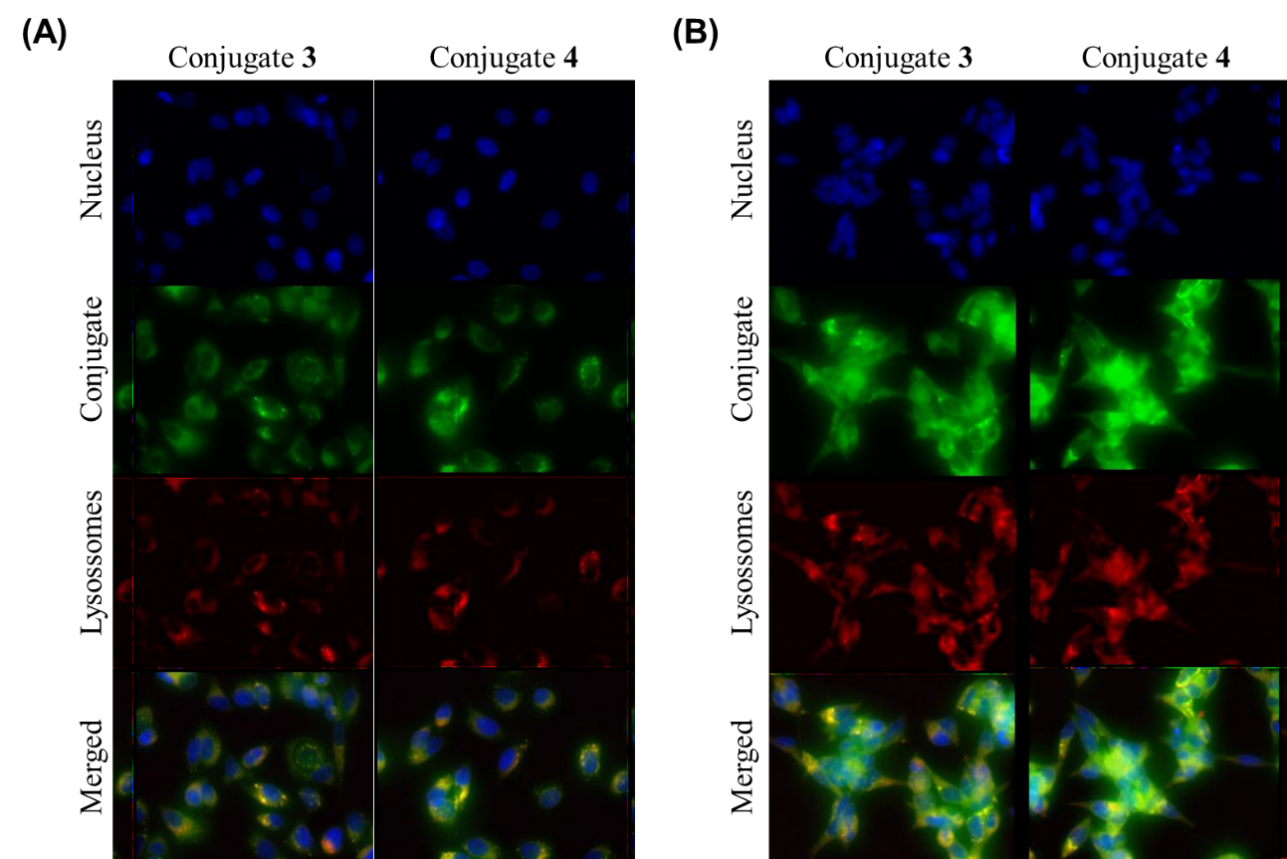


**Figure S10.** Intracellular localization of androgen-BODIPY conjugates **3** and **4** in (A) PC3 and (B) LNCaP prostate cancer cell lines after 6h incubation. PC3 and LNCaP cells were seeded for 24h in complete RPMI medium (with Phenol red) and then medium was replaced with RPMI without phenol red and 50 μM of conjugates **1** and **2**. After fixation with formaldehyde and permeabilization with Triton X-100, cells were stained with Hoechst 33258 (nucleus marker) and LAMP-2 (lysosome marker). Cells were visualized on inverted microscope with amplification 400x and images of Hoechst channel were acquired with UV excitation (blue), LAMP-2 channel with green excitation (red) and conjugate channel with blue excitation (green). The presented fluorescence images were treated using Image J software.

**Cell viability assays**

The cytotoxic potential of the conjugates in the different human cancer and normal cell lines was performed by the MTS assay. This assay is based on MTS reagent biorreduction by intracellular dehydrogenases of metabolically active cells into formazan (soluble product), being the amount of formazan measured at 490 nm directly proportional to the number of viable cells. Based on previously not published studies, a 50μM concentration of each conjugate was used and MTS assay was performed after 6h and 24h exposition in the cancer cell lines as well in normal human fibroblasts.

Estradiol-BODIPY analogues (EE2-C8 and 11β-OMe) were tested in breast cancer cell lines, MCF-7 and MDA-MB-231 and in normal cells, Fibroblasts. **(Figure S11 (A), (B), (C)**) Androgen-BODIPY analogues were tested in prostate cancer cell lines, PC-3 and LNCaP and also in normal cells, Fibroblasts **(Figure S11 (D), (E), (F)**)**.** No antiproliferative effect was observed for all steroid-BODIPY conjugates and both exposure periods in normal cells and in all of types of cancer cells, except for the HA-4199 and HA-4200 androgen-BODIPY conjugates, that apparently lead to a very slight decrease of viability in LNCaP cells. In general, these results showed that all conjugates were non-toxic to cancer and normal cells, enabling their application for certain cancer therapies, namely PDT.


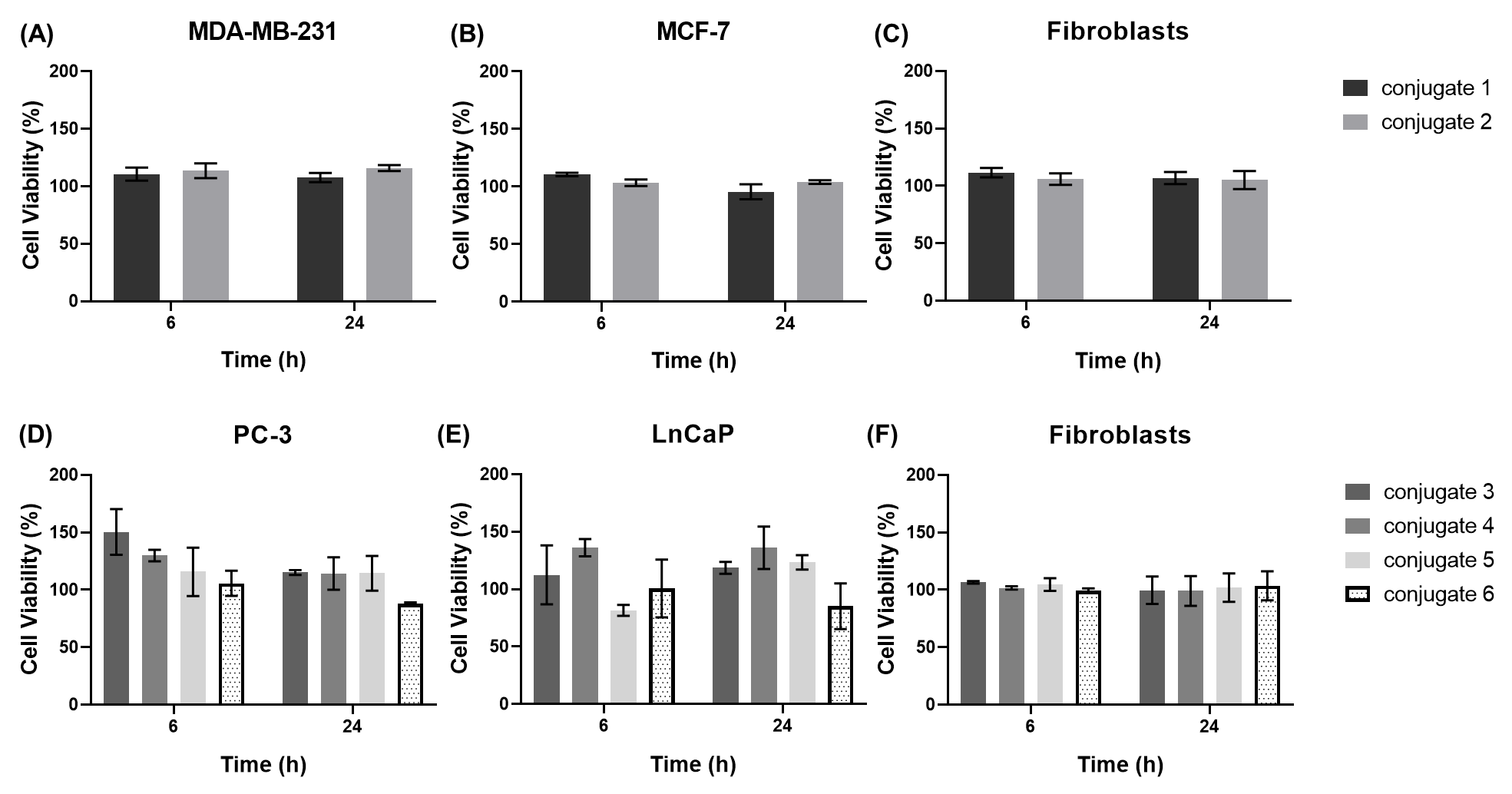
**Figure S11.** Cell viability of estradiol-BODIPY conjugates **1** and **2** in breast cancer cells (A) MDA-MB-231, (B) MCF7 and (C) normal dermal Fibroblasts, or androgen-BODIPY conjugates **3-6** in prostate cancer cells (D) PC-3, (E) LNCaP and (F) normal dermal fibroblasts. Cells were incubated with 50 μM of steroid- BODIPY conjugates 1-6 for 6h and 24h and cell viability was determined by MTS. Data was normalized against the control (0.5% (v/v) DMSO) and expressed as the mean ± SEM of two independent assays.

Irradiation with green visible light with a laser diode intensity of 2.45 W/cm^2^ was tested in breast cancer cell line MCF-7, in prostate cancer cell line PC-3 and in normal dermal Fibroblasts. **(Figure S12**) to evaluate a possible toxic effect on cells. No antiproliferative effect was observed, suggesting that irradiation of the cells alone at the parameters used does not produce a toxic effect on the cell lines.


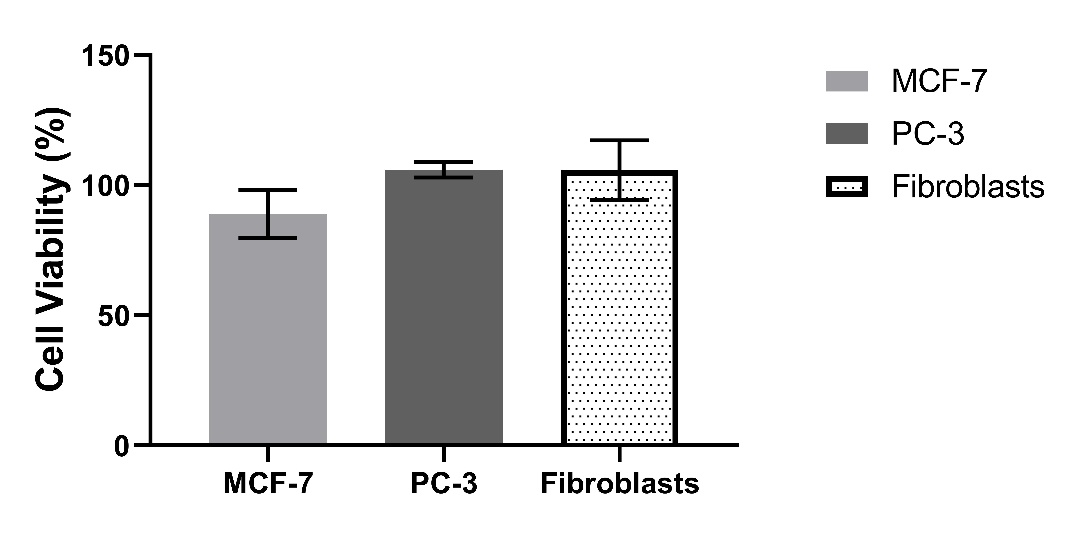


**Figure S12.** Cell viability of MCF-7, PC3 and normal dermal fibroblasts via MTS 24h after visible irradiation for 60 seconds. Data was normalized against the control (correspondent cells without irradiation) and expressed as the mean ± SEM of two independent assays.

**Visible light irradiation**

Preliminary tests were performed to choose the conditions of this study: conjugate concentration, laser diode intensity and the exposition time (see **Figure S11**). PC-3 cells were incubated with 50 μM of androgen-BODIPY conjugate **4** and irradiated with three different laser diode intensities (2.45, 3.12 and 3.78 W/cm^2^) for 60 seconds. These results demonstrated that cell viability decreases approximately 70% after irradiation for all tested LDI. Nevertheless, despite these results, the lowest LDI (2.45 W/cm^2^) was chosen. The concentration of conjugates used in the next studies was 25 μM because of conjugate saturation with highest concentration of 50 μM.

**Figure S13.** Prostate cancer cell death following PDT induced by visible light and 50 μM of androgen-BODIPY conjugate **4** as photosensitizer. Cell viability via MTS assay in PC-3 cell line 24 h after visible irradiation with three different LDI (2.45, 3.12 and 3.78 W/cm^2^) for 60 seconds. Data was normalized against the control (0.5% (v/v) DMSO) and expressed as the mean ± SEM of two independent assays.
